# Red flowers differ in shades between pollination systems and across continents

**DOI:** 10.1093/aob/mcaa103

**Published:** 2020-06-01

**Authors:** Zhe Chen, Yang Niu, Chang-Qiu Liu, Hang Sun

**Affiliations:** 1 CAS Key Laboratory for Plant Diversity and Biogeography of East Asia, Kunming Institute of Botany, Chinese Academy of Sciences, Kunming, Yunnan, China; 2 University of Chinese Academy of Sciences, Beijing, China; 3 Center for Gardens and Horticultural Studies, Guangxi Institute of Botany, Guangxi Zhuang Autonomous Region and Chinese Academy of Sciences, Guilin, Guangxi, China

**Keywords:** Bee, bird, pollinator, colour vision, floral colour, flower evolution, plant-animal interaction, pollination syndrome

## Abstract

**Background and Aims:**

Floral colour is a primary signal in plant–pollinator interactions. The association between red flowers and bird pollination is well known, explained by the ‘bee avoidance’ and ‘bird attraction’ hypotheses. Nevertheless, the relative importance of these two hypotheses has rarely been investigated on a large scale, even in terms of colour perception *per se*.

**Methods:**

We collected reflectance spectra for 130 red flower species from different continents and ascertained their pollination systems. The spectra were analysed using colour vision models for bees and (three types of) birds, to estimate colour perception by these pollinators. The differences in colour conspicuousness (chromatic and achromatic contrast, purity) and in spectral properties between pollination systems and across continents were analysed.

**Key Results:**

Compared with other floral colours, red flowers are very conspicuous to birds and much less conspicuous to bees. The red flowers pollinated by bees and by birds are more conspicuous to their respective pollinators. Compared with the bird flowers in the Old World, the New World ones are less conspicuous to bees and may be more conspicuous not only to violet-sensitive but also to ultraviolet-sensitive birds. These differences can be explained by the different properties of the secondary reflectance peak (SP). SP intensity is higher in red flowers pollinated by bees than those pollinated by birds (especially New World bird flowers). A transition from high SP to low SP in red flowers can induce chromatic contrast changes, with a greater effect on reducing attraction to bees than enhancing attraction to birds.

**Conclusions:**

Shades of red flowers differ between pollination systems. Moreover, red bird flowers are more specialized in the New World than in the Old World. The evolution towards colour specialization is more likely to result in higher efficiency of bee avoidance than bird attraction

## INTRODUCTION

Colour is an important visual signal in plant–animal interactions. The colour of flowers (and sometimes floral accessories) is a primary signal in pollinator attraction (Schaefer *et al.*, 2004; Schaefer and Ruxton, 2011) and is an important dimension of pollination syndromes (Fenster *et al.*, 2004). Red floral colour has long been regarded as an important aspect of the bird pollination syndrome, combined with other characters such as tubular floral shape, copious nectar and absence of odour (Willmer, 2011). For example, Pickens (1930) found that more than 50 % of hummingbird-pollinated flowers are red or orange in the eastern USA.

Generally, signals are thought to be evolutionary results of compromise; they should be received by the intended recipients efficiently and readily while being inconspicuous to unintended recipients that have the potential to reduce the fitness of the signal senders (Endler, 2000). Two hypotheses have been proposed to explain the association between red flowers and bird pollination: bird attraction and bee avoidance (Grant, 1966; Raven, 1972). The bird attraction hypothesis is based on the fact that birds have red photoreceptors. Birds are usually tetrachromats, possessing four kinds of single cones [based on short-wavelength-sensitive opsins (SWS1 and SWS2), medium wavelength-sensitive opsin (MWS) and long-wavelength-sensitive opsin (LWS); Hart and Hunt, 2007]. Their red photoreceptors (LWS cones) have maximum absorbance wavelengths (*λ*_max_) at 601–620 nm (Hart and Hunt, 2007) and account for most of the photoreceptors (single cones; Hart, 2001*a*) in the retina. Therefore, avian red photoreceptors are sensitive to the colours that humans define as red, and it is believed that red flowers are more conspicuous and attractive to birds than flowers of other colours (Grant, 1966; Chittka and Waser, 1997; Herrera *et al.*, 2008).

The bee avoidance hypothesis is based on at least three facts. First, although bees do visit flowers pollinated by birds, their pollination efficiency is generally lower than that of bird pollinators (Castellanos *et al.*, 2003; Thomson and Wilson, 2008; Bergamo *et al.*, 2016; Krauss *et al.*, 2017). Second, their robbing behaviour may further reduce plant fitness (Irwin *et al.*, 2010; Rojas-Nossa *et al.*, 2016; but see Maloof and Inouye, 2000). Third, their ability to process red signals visually is not very good (Chittka and Wells, 2004) because they do not have red photoreceptors in their eyes. Bees have trichromatic colour vision based on three classes of photoreceptors (UV, blue and green photoreceptors) whose maximum sensitivities are in the UV, blue and green (*λ*_max_ ≈ 540 nm for the bumblebee *Bombus terrestris*; Skorupski *et al.*, 2007) regions, respectively. This colour vision is highly conservative in hymenopterans (Peitsch *et al.*, 1992; Dyer *et al.*, 2015), making them generally relatively weak at processing red signals chromatically. Therefore, bird-pollinated red flowers may achieve higher fitness by remaining less attractive to relatively inefficient visitors and flower parasites (nectar robbers).

These two hypotheses are not mutually exclusive, but their relative importance in the evolution of red flowers may not be equal. Some earlier studies implied that bird attraction could be more important (Pickens, 1930; Grant, 1966; Vickery, 1992), whereas a few more recent works support the bee avoidance hypothesis (Altshuler, 2003; Lunau *et al.*, 2011; Bergamo *et al.*, 2016). However, although behavioural experiments are essential to test these hypotheses (whether colour signal transfers to fitness difference), the estimation of colour perception *per se* may provide valuable clues. Actually, the two hypotheses may be associated with different expectations in terms of colour perception and these have seldom been tested on a large scale. If bird attraction is more important for the evolution of bird-pollinated flowers, these flowers may not necessarily be difficult for bees to detect. In contrast, if bee avoidance is more important, bird-pollinated red flowers may not necessarily be very conspicuous to birds.

Another fact that has received less attention is that red flowers also occur in other pollination systems. Many flowers pollinated by butterflies (Johnson and Bond, 1994; Willmer, 2011) and beetles in some areas are characterized by a red colour (Dafni *et al.*, 1990). In addition, although scarce, there are indeed some plants pollinated by bees that produce red flowers (e.g. *Onosma confertum*; Chen *et al.*, 2020). There may be some common associations in terms of colour perception between different pollinators and their floral colours.

**Fig. 1.**
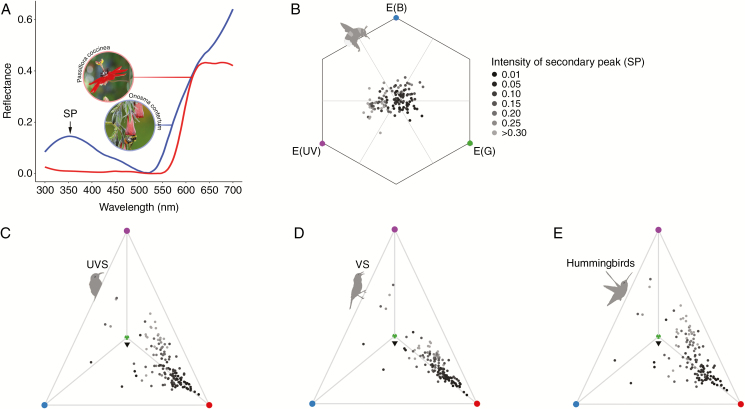
Characteristics of floral reflectance spectra and colour loci of 130 red flower species in the study according to different colour vision models. (A) Reflectance spectra of *Onosma confertum* and *Passiflora coccinea*, as representatives of red flowers with and without an SP. (B) Colour loci in a CH model for bees; purple, blue and green vertices represent the maximum signals (1) in UV, blue and green photoreceptors. E, receptor signal. (C–E) Colour loci in the tetrahedron colour spaces for UVS birds, VS birds and hummingbirds. The inverted triangles in the centres refer to the origin points; purple, blue, green and red vertices represent the maximum signals (relative quantum catches) (1) in SWS1, SWS2, MWS and LWS receptors. Point brightness represents SP intensity of each floral spectrum, with darker points corresponding to spectra with lower SPs.

More importantly, the term ‘red’ has been defined based on the perception of humans, although it is a colour complex that may contain several colours in the pollinators’ colour vision (and also in human colour vision *per se*). Based on reflectance properties, Chittka and Waser (1997) categorized human-detected red floral reflectance into three groups: flowers that only exhibit great reflectance above 600 nm; flowers that also have a secondary reflectance peak (SP) in the blue region; and flowers that have an SP below 400 nm (Fig. 1A). Because of the spectral differences in the short wavelengths, these flower colours certainly appear different to pollinators that possess a UV receptor, such as bees (Chittka and Waser, 1997; Spaethe *et al.*, 2001; Martínez-Harms *et al.*, 2010). Lunau *et al.* (2011) discovered different UV reflectance properties between hummingbird- and bee-pollinated red flowers, and, behaviourally, bees prefer UV-reflecting more than UV-absorbing red. It seems reasonable to predict that the SP is important for pollinators that lack red photoreceptors in detecting red targets (Chittka *et al.*, 1994; Martínez-Harms *et al.*, 2010; Lunau *et al.*, 2011). However, its influence on the colour perception of pollinators possessing red photoreceptors is still not very clear.

Bird pollination occurs in many angiosperm lineages. More than one thousand bird species participate in pollination, which involves several thousand plant species in ~500 genera (Sekercioglu, 2006; Pauw, 2019). Bird pollinators deeply affect the evolution of flower traits in various aspects (Cronk and Ojeda, 2008; Krauss *et al.*, 2017). Based on their spectral sensitivity to short-wavelength light, birds can be further divided into two colour vision types. The ultraviolet-sensitive (UVS) type has maximum sensitivity at 355–380 nm, whilst the violet-sensitive (VS) type is sensitive at 402–426 nm (Hart, 2001*b*; Ödeen *et al*., 2009). More interestingly, there is a phylogenetic and geographical pattern associated with avian pollinators with these two different visual systems. Studies on the amino acid sequence of SWS1 showed that sunbirds (~132 species; Nectariniidae), the primary avian pollinators in Asia and Africa, are UVS (*λ*_max_ = 371 nm), while hummingbirds (~340 species; Trochilidae), now exclusive to the Americas, are VS (*λ*_max_ = 406 nm; Cronk and Ojeda, 2008; Ödeen and Håstad, 2010; Krauss *et al.*, 2017; but see Herrera *et al.*, 2008). This difference in birds’ colour vision has rarely been considered in previous studies on floral colour evolution (but see Burd *et al.*, 2014). Comparisons of bird-pollinated red flowers across continents may shed light on the interaction between floral colour and avian pollinators.

In this study, we collected floral reflectance data for red flowers native to different continents and classified them by pollination systems. Floral colours were then analysed using different colour vision models to estimate floral colour conspicuousness (in terms of chromatic and achromatic contrast against the background, and purity) that may influence pollinators’ detection of flowers and their behaviour. We undertook comparative analyses and asked the following three questions.

(1) Is the red floral colour, compared with other colours, more conspicuous to birds or/and less conspicuous to bees?

(2) Is there any colour difference between bee- and bird-pollinated red flowers (hereafter bee and bird flowers, respectively), or more generally, between flowers pollinated by animals with different colour vision [i.e. with red photoreceptors (R+) or without them (R−)]? Considering the absence of red photoreceptors, we predict that red flowers pollinated by R− animals (such as bees) tend to have high SP intensity, whereas flowers pollinated by R+ animals (e.g. birds) may not necessarily have this characteristic.

(3) For bird flowers, is there any colour difference between bird-pollinated red flowers from the Old World (OW) and the New World (NW)? And how differently are they perceived by birds with different colour vision? If the red colour of bird flowers has evolved mainly to avoid bees (with relatively conservative colour vision), there may be convergent evolution leading to similar colours that perform best in bee avoidance, regardless of how they are perceived by birds, whereas if flowers have evolved to attract birds there may be a colour divergence between continents, optimizing conspicuousness to different birds (with UVS and VS vision).

## MATERIALS AND METHODS

### Colour definition and data collection

Different names are used to describe various shades of human-subjective red colours, such as scarlet, cardinal and vermilion. Despite all of them having primary reflectance at long wavelengths (Fig. 1A), it is not easy to give a strict definition of red, as it depends on the criteria and specific individual psychophysics. Chittka and Waser (1997) used 611 nm as the boundary between human red and orange. However, given that bird flowers also include some orangish colours (e.g. *Campsis radicans*), in the present study we extended this boundary to 560 nm (also used by Reisenman and Giurfa, 2008). Therefore, the term ‘red’ here includes different shades of red and orange colours that mainly reflect at long wavelengths (>560 nm). Spectral curves were collected from direct measurement (*N* = 76), the Floral Reflectance Database (FReD, http://www.reflectance.co.uk, *N* = 25) (Arnold *et al.*, 2008) and the literature (*N* = 29, detailed in Supplementary Data Table S1 and Fig. S1). There was no personal bias in the process of data collection. We tried to collect as much data as possible to cover a relatively large range at both phylogenetic and geographical levels. These 130 red flower species belonged to 104 genera in 53 families (detailed in Supplementary Data Table S1), including both monocotyledons and eudicotyledons, and are widely spread in terms of phylogeny (including commelinids, fabids, malvids, campanulids and lamiids). In addition, their geographical distributions involve all the continents but Antarctica, which were determined based on the literature. We also collected floral reflectance data for non-red colours personally (*N* = 30) and from the FReD (*N* = 282) for comparison.

A spectrometer (USB 2000+, Ocean Optics) equipped with a UV-VIS light source (DH2000, Ocean Optics) was used to measure floral reflectance, using a pressed pellet of barium sulphate as the white standard. The fibre-optic probe was fixed at 45° to the measuring spot. At least three fresh flowers from different individual plants were used to obtain reflectance curves between 300 and 700 nm, and the average was used.

Sampled plants were assigned to a specific pollination system (i.e. pollinated by bees, hummingbirds, sunbirds, butterflies or beetles) mainly based on the available literature, involving pollinator efficiency, visitation proportion and/or pollinator behaviour (i.e. contact with reproductive organs). When a particular group of animals contributed >70 % of total pollen transfer, fruit/seed set or visitations, this group was categorized as the main pollinator(s). Visitor behaviour is also considered to determine effective pollinators and to exclude visitors with no or little effect on pollination (Fenster *et al.*, 2004). Very few species (*N* = 13) were reported as being pollinated by two or more pollinator groups, and these were excluded from some analyses (see details in the descriptions of each analysis given below). When the literature was limited, pollinators were determined by direct observation for 2–5 d of fine weather (between about 0800 and 1700 h) in the wild (*N* = 14) or botanic gardens (*N* = 2, butterfly-pollinated) for each of these species in successive years. In a few cases (*N* = 7) pollinators were inferred mainly based on pollination syndromes. Although the presence of pollination syndromes has been debated (Fenster *et al.*, 2004; Ollerton *et al.*, 2009), pollination syndromes do provide clues to infer the potential pollinator(s) when other information is not available. Pollination evidence from congeners with very similar floral traits were considered as well. Here we considered the main pollinators as pollinator groups while filtering out the potential noise caused by illegal visitors or visitors with low efficiency. For instance, although typical bird flowers may also be visited by bees, their floral traits are mainly adapted to the most efficient pollinators, the birds. Details about the plants are presented in Supplementary Data Table S1.

### Colour vision models

Colour vision models were used to estimate colour perception by different kinds of pollinators. These models have been established on the basis of animal anatomical and physiological properties, combining knowledge from behavioural and/or psychophysical experiments (Kelber and Osorio, 2010; Renoult *et al.*, 2017). Here, two widely used colour vision models were employed: the colour hexagon model (CH model; Chittka, 1992) and the receptor noise-limited model (RNL model, Weber fraction version; Vorobyev *et al.*, 2001). The CH model is exclusive to trichromatic Hymenoptera; it produces a two-dimensional equilateral hexagon space in which colour perception by bees can be analysed and visualized (Fig. 1B). Colour (chromatic) difference in the CH model is determined by the Euclidean distance between colour loci (in CH units); a colour locus close to the origin (we placed the green leaf background here) is similar to the background and is difficult for bees to detect by chromatic mechanisms (Chittka *et al.*, 1994; Spaethe *et al.*, 2001). The RNL model assumes that receptor noise limits colour discrimination; this is applicable to both tri- and tetrachromatic colour vision. Colour difference in the RNL model is given in ‘just noticeable difference’ (JND) units. Although the RNL model was originally established for evaluating the perception of small colour differences near the detection threshold, its application to the evaluation of perception of large colour differences has proved successful in research involving bees (Hempel de Ibarra *et al.*, 2001; Niggebrügge and Hempel de Ibarra, 2003) and birds (Stobbe and Schaefer, 2008; Cazetta *et al.*, 2009).

Receptor sensitivity functions and receptor noise values are the key parameters of the models. The Weber fraction, ω, was used as a substitute for the noise value (in the RNL model) as it refers to the minimum stimuli difference/change that is perceptible, and any difference smaller than the Weber fraction is unnoticeable and just perceived as noise. Receptor sensitivity functions of bumblebees (*Bombus terrestris dalmatinus*) were obtained from Skorupski *et al.* (2007), and the receptor noise values of UV, blue and green receptors (0.74, 0.67 and 0.61, respectively) from Skorupski and Chittka (2010). For birds, average receptor sensitivity functions for UVS and VS birds based on the model in Endler and Mielke (2005) were used. We used 0.1 as the Weber fraction for avian LWS receptors (Maier, 1992). Consequently, the corresponding Weber fraction values for UVS birds were 0.2, 0.1414, 0.1414 and 0.1, and for VS birds they were 0.1414, 0.1414, 0.1414 and 0.1. Although hummingbirds’ colour vision was classified as the VS type based on the sequence of SWS1 opsins (Ödeen and Håstad, 2010), the only empirical study based on electroretinogram recordings of a hummingbird (*Sephanoides sephaniodes*) showed a *λ*_max_ at ~370 nm. This groups hummingbirds in the UVS type (Herrera *et al.*, 2008; but see Ödeen and Håstad, 2010). As a reference, receptor sensitivity functions of this hummingbird were also used (Herrera *et al.*, 2008), with 0.2, 0.1414, 0.1414 and 0.1 as their corresponding Weber fraction values. Details of the models and calculations used are presented in Supplementary Data Methods S1.

For visualization, colour spaces were constructed for bees (CH model) and birds (tetrahedron model; Goldsmith, 1990), using the R package pavo (Maia *et al.*, 2013), in which colour can be presented as a point (colour locus; Fig. 1B–E). For the origin (background) of each colour space, an average of 230 leaf reflectances was used (Chittka *et al.*, 1994).

### Colour conspicuousness of flowers

Colour conspicuousness is largely determined by the contrast between a target and its background, and this greatly affects visual attractiveness to the signal receivers (Carter and Carter, 1981; Schmidt *et al.*, 2004). It includes two aspects: chromatic contrast and achromatic contrast. For bees, visual attractiveness may also be affected by purity (Lunau *et al.*, 1996; Rohde *et al.*, 2013).

‘Chromatic contrast’ describes the colour contrast that excludes brightness information. For bees, this parameter was calculated in both the CH and the RNL model; for birds, it was calculated in the RNL model only. A larger chromatic contrast value indicates a stronger flower–leaf contrast in colour, which facilitates detection.

‘Achromatic contrast’ refers to the brightness difference between a target and its background. Here, it was calculated following a previous study by Papiorek *et al.* (2015), dividing flower–leaf background contrast in green photoreceptors (for bees) or in double cones (for birds) by the Weber fractions of corresponding photoreceptors. For birds, the double-cone spectral sensitivity functions of blue tit (*Cyanistes caeruleus*; Hart *et al.*, 2000), domestic chicken (*Gallus gallus*; Osorio *et al.*, 1999) and a hummingbird (*Sephanoides sephaniodes*; Herrera *et al.*, 2008) were used for UVS birds, VS birds and hummingbird models, respectively, and we assumed their corresponding Weber fraction values are all 0.05 (Siddiqi *et al.*, 2004).

‘Purity’ refers to the saturation of colours, or how ‘vivid’ the colours were perceived to be by animals. At present the effect of purity on visual attraction has been verified only in bees, which prefer colours with higher purity (Rohde *et al.*, 2013). This parameter was estimated in the CH model, dividing the flower–background distance by the corresponding monochromatic light–background distance (Lunau *et al.*, 1996).

### Comparisons between red and other floral colours

Colour conspicuousness of red flowers (*N* = 130) and six other colour categories (blue, *N* = 41; pink, *N* = 38; purple, *N* = 21; violet, *N* = 38; white, *N* = 86; yellow, *N* = 88; Supplementary Data Fig. S2) were compared to examine whether human red is indeed the most conspicuous colour to birds or/and the least conspicuous colour to bees (phylogenetic ANOVA, detailed below in the Phylogenetic constraints section).

### Shades of red in different pollination systems and on different continents

Colour conspicuousness between bee flowers (*N* = 10), OW bird flowers (*N* = 37) and NW bird flowers (*N* = 47) were compared from the perspectives of different pollinators to explore the potential differences in red coloration between pollination systems (phylogenetic ANOVA). Given the potential difference between bird flowers within the OW, this was further divided into Asia and Africa to make comparisons between different continents (Asia, Africa and the Americas; phylogenetic ANOVA).

### Secondary peaks in red flowers

We assumed that the difference in colour conspicuousness between flower groups largely stems from the difference in their spectral properties, especially the intensity of SPs. To examine this, we calculated a ratio, *k*, between the height of the SP and the height of the whole spectrum, which was used to quantify the intensity of an SP and to perform the subsequent analyses.

#### SP intensity pattern

First, the intensity of the SP in red flowers was compared between pollination systems (bees and OW and NW bird flowers). Then, to explore a more general association between spectral characteristics and animal colour vision properties, red flowers (*N* = 110; species whose pollinators lack clear colour vision information were excluded, e.g. certain butterflies or those flowers with a mix of different pollinator groups) were divided into two groups: those pollinated by animals equipped with red photoreceptors (R+ animals, such as birds, some beetles and butterflies) and the those by animals without such receptors (R− animals, i.e. bees; see Supplementary Data Table S1 for details). The SP intensity of these two flower groups was compared (phylogenetic ANOVA).

#### Influence of SP on conspicuousness

To explore the effect of SP on floral conspicuousness, we used phylogenetic linear regression analyses (detailed below in the Phylogenetic constraints section) for each conspicuousness parameter against SP intensity.

### Phylogenetic constraints

To control the potential influence of phylogeny on the evolutionary patterns of colour phenotype, we performed phylogenetic ANOVAs using the function phylANOVA (with 1000 simulations and Holm’s method for *P*-value adjustment) in the package phytools (Revell, 2011). For phylogenetic regression, the function pgls in the package caper was used (Orme *et al.*, 2018). Phylogenetic constraints were adjusted by estimating the maximum likelihood of Pagel’s *λ* (Pagel, 1999). The phylogenetic tree of our sample species (Supplementary Data Figs S1 and S2) was constructed using the phylo.maker function implemented in R package V.PhyloMaker (Jin and Qian, 2019), which uses a dated mega-tree derived from the research of Zanne *et al.* (2014) and Smith and Brown (2018) as a backbone. All the 85 families and 282 (95.3 %) out of the 296 genera in our study were found in this mega-tree. For the species absent from the mega-tree, they were added using the Scenario 3. All statistical analyses were conducted in R 3.5.0 (R Core Team, 2018).

## RESULTS

### General pattern

Of the 130 red flower species (belonging to 104 genera in 53 families) that we investigated, there were ten (7.7 %, one from the NW and the others from the OW) pollinated by bees, 37 (28.5 %, all native to the OW) pollinated by sunbirds (and by several other OW birds), 47 (36.2 %, all native to the NW) pollinated by hummingbirds, five (3.8 %) by beetles, 18 (13.8 %) by butterflies and 13 (10 %) by more than one pollinator functional group (see details in Supplementary Data Table S1). Our data are not seriously influenced by redundancy from the perspective of taxonomy: most genera (*N* = 90, 86.5 %) in our data contain one plant species. The biggest genus here, *Rhododendron*, has seven species. The other genera contain no more than three species each. About half of the families contain one genus (*N* = 28, 52.8 %). Only six families contain more than three genera (Fabaceae, *N* = 7; Solanaceae, *N* = 7; Bignoniaceae, *N* = 5; Gesneriaceae, *N* = 5; Lamiaceae, *N* = 5; Asteraceae, *N* = 4), which should reflect the sizes of these families. In addition, the phylogeny-informed method used in the following analyses, which, considering the influence of phylogeny on analysis, can to some extent mitigate the potentially slight redundancy and imbalance.

In the bees’ colour space (CH model; Fig. 1B), floral colour loci were generally clustered around the central zone, so may be perceived as colours very similar to the leaf background (the origin) by bees. To birds (in the tetrahedron models; Fig. 1C–E), the flower colour loci mainly clustered along the axis of the red photoreceptors. Colour loci showed a more dispersed distribution in the model spaces for UVS birds (Fig. 1C) and hummingbirds (Fig. 1E) than VS birds (Fig. 1D), which may reflect the inherent difference between these colour vision systems.

### Comparison between red and other floral colours

**Fig. 2.**
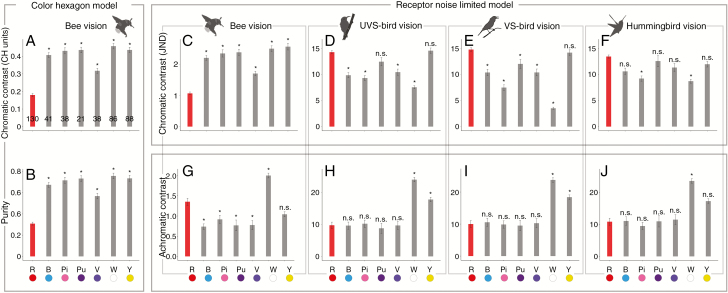
Comparisons of parameters of colour conspicuousness between human-subjective red (R) and the other six floral colours according to different colour vision models. The six other floral colours are blue (B), pink (Pi), purple (Pu), violet (V), white (W) and yellow (Y). The numbers at the bottom of the columns in (A) are sample sizes. The RNL model was used for both bees and birds, and the CH model was used in addition for bees. (A, B) Chromatic contrast and purity in bees’ vision according to the CH model. (C–F) Chromatic contrast in bees’, UVS birds’, VS birds’ and hummingbirds’ vision according to the RNL model. (G–J) Achromatic contrast in bees’, UVS birds’, VS birds’ and hummingbirds’ vision. phylANOVAs (with 1000 simulations and Holm’s method for *P*-value adjustment) were used. Values are shown as mean ± s.e. *P < 0.05; n.s., not significant.

Chromatically, to bees, human red floral colour appears less conspicuous on the leaf background than the other six floral colours we considered. Red flowers showed a much lower chromatic contrast (*P* values < 0.05 for all; Fig. 2A, C) and a much lower purity (*P* values < 0.05 for all; Fig. 2B). For birds (UVS birds, VS birds, and hummingbirds), however, red flowers showed the highest chromatic contrasts among the various floral colours (*P* < 0.05 for all, with the exception of yellow and purple, violet and blue on some occasions; Fig. 2D–F). Achromatically, to bees, red exhibited lower contrast than white and higher contrast than the other colours (except yellow; Fig. 2G). To birds, red had lower achromatic contrast than white and yellow usually, but showed no significant differences from other colours (Fig. 2H–J).

### Colour conspicuousness of red flowers

#### Chromatic contrast

**Fig. 3.**
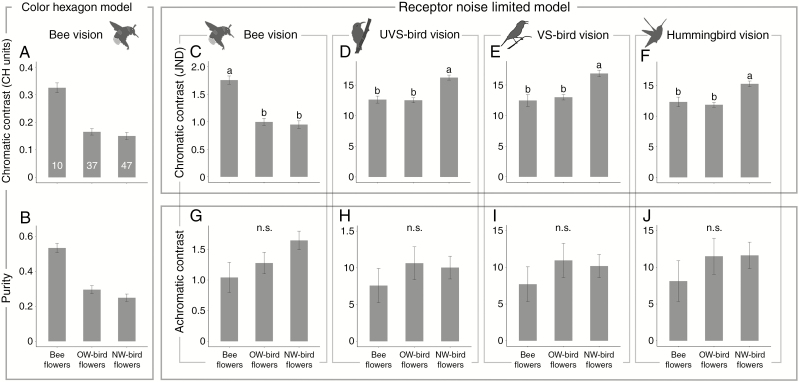
Comparisons of parameters of colour conspicuousness between red flowers pollinated by bees, OW birds and NW birds according to different colour vision models. The RNL model was used for both bees and birds, and the CH model was used in addition for bees. (A, B) Chromatic contrast and purity in bees’ vision according to the CH model. (C–F) Chromatic contrast in bees’, UVS birds’, VS birds’ and hummingbirds’ vision according to the RNL model. (G–J) Achromatic contrast in bees’, UVS birds’, VS birds’ and hummingbirds’ vision. phylANOVAs (with 1000 simulations and Holm’s method for *P*-value adjustment) were used. Values are shown as mean ± s.e. Different letters above the error bars indicate significant differences at 0.05 level (n.s., not significant). The numbers at the bottom of the columns in (A) are the sample sizes.

To bees, chromatic contrast of bee-pollinated red flowers was higher than that of bird-pollinated flowers under both the CH model (*P* = 0.003 for both comparisons with OW and NW bird flowers; Fig. 3A) and the RNL model (*P* = 0.003 for both comparisons with OW and NW bird flowers; Fig. 3C). To birds (UVS birds, VS birds and hummingbirds), however, the NW bird flowers exhibited higher chromatic contrast than not only the bee flowers (*P* = 0.012, 0.008 and 0.036, respectively; Fig. 3D–F) but also the OS bird flowers (*P* = 0.003, 0.006 and 0.003, respectively; Fig. 3D–F). The better performance of the NW bird flowers in UVS birds’ vision was unexpected. The difference between OS bird flowers and bee flowers was not significant (Fig. 3D–F).

#### Purity

Purity was higher in bee flowers than in the OW and NW bird flowers (each *P* = 0.003), and it was similar between the two groups of bird flowers (Fig. 3B).

#### Achromatic contrast

For any vision system (bees’, UVS-birds’, VS birds’ and hummingbirds’ vision), there were no significant differences in achromatic contrast between any floral groups (Fig. 3G–J).

#### Geographical pattern

**Fig. 4.**
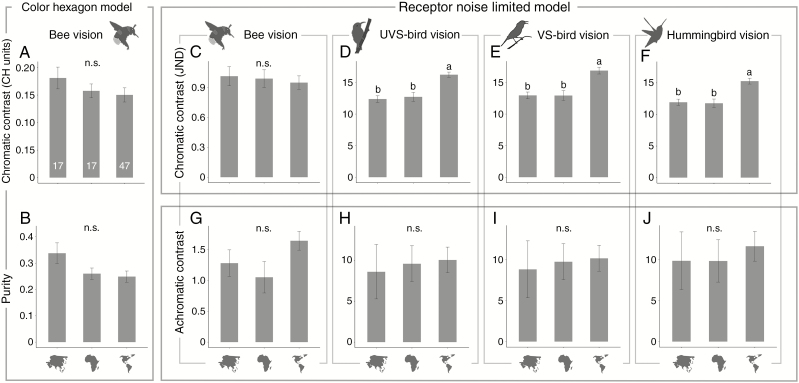
Comparisons of parameters of colour conspicuousness between bird-pollinated red flowers from Asia, Africa and the Americas (indicated by small maps under the columns) according to different colour vision models. The RNL model was used for both bees and birds, and the CH model was used in addition for bees. (A, B) Chromatic contrast and purity in bees’ vision according to the CH model. (C–F) Chromatic contrast in bees’, UVS birds’, VS birds’ and hummingbirds’ vision according to the RNL model. (G–J) Achromatic contrast in bees’, UVS birds’, VS birds’ and hummingbirds’ vision. phylANOVAs (with 1000 simulations and Holm’s method for *P*-value adjustment) were used. Values are shown as mean ± s.e. Different letters above the error bars indicate significant differences (n.s., not significant). The numbers at the bottom of the columns in (A) are sample sizes.

Further comparisons of bird flowers across the three continents (Asia, Africa and the Americas) from the perspectives of birds (UVS birds, VS birds and hummingbirds) showed that the NW bird flowers had higher chromatic contrast than the Asian (*P* = 0.006, 0.032 and 0.026, respectively) and African ones (*P* = 0.003, 0.015 and 0.009, respectively; Fig. 4D–F). There was, however, no significant difference between Asia and Africa (Fig. 4D–F).

### Effect of SP in red flowers and its colour properties on perception

**Fig. 5.**
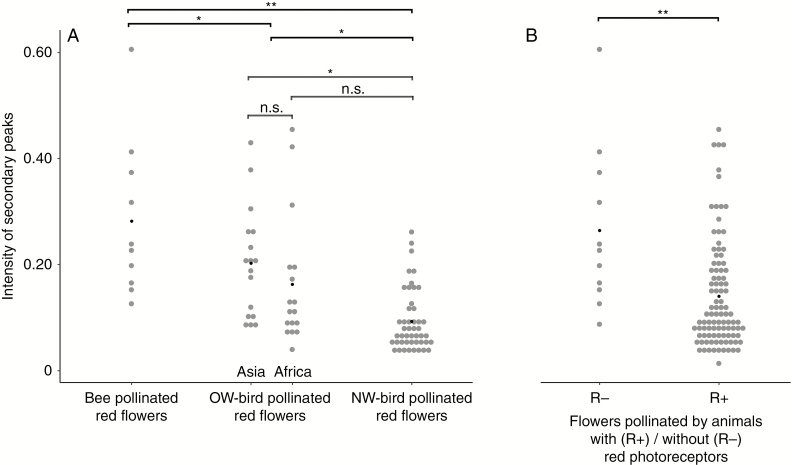
Intensity of SPs in different red floral groups. (A) SP intensity of red flowers pollinated by different pollinators (bees and birds on different continents). (B) SP intensity of red flowers pollinated by animals with (R+) and without (R−) red photoreceptors. The light grey points show the SP intensity of red flowers of different species. Dark points show the mean values of each group. phylANOVAs (with 1000 simulations and Holm’s method for *P* value adjustment) were used. *P < 0.05; **P < 0.01; n.s., not significant.

The intensity of the SP was higher in bee-pollinated red flowers than in the OW and NW bird flowers (*P* = 0.022 and 0.003, respectively; Fig. 5A), and higher in OS bird flowers than in NW bird flowers (*P* = 0.022; Fig. 5A). The OW was further divided into Asia and Africa. Asia showed higher SPs than the Americas (*P* = 0.030; Fig. 5A). However, Africa showed no difference from Asia or the Americas (*P* = 0.363 and 0.082, respectively).

Flowers were then divided into two groups depending on whether their pollinators had a red receptor or not. Comparative analysis showed that flowers pollinated by R− animals (bees and *Danaus* butterflies) tended to have a higher SP, whereas those pollinated by R+ animals (such as beetles, some butterflies, and birds) tended to have a lower or no SP (*P* = 0.005; Fig. 5B).

**Fig. 6.**
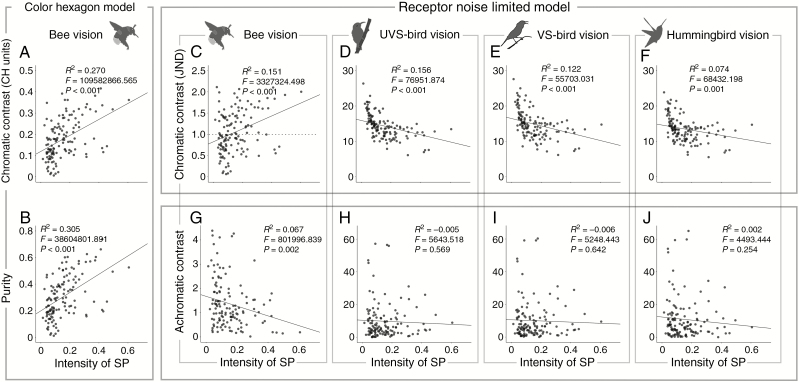
Relationship between floral parameters and the intensity of SPs. Relationship between SP intensity and (A, B) chromatic contrast (A) and purity (B) in bees’ vision according to the CH model; (C–E) chromatic contrast in bees’ (C), UVS birds’ (D), VS birds’ (E) and hummingbirds’ (F) vision according to the RNL model; and (G–J) achromatic contrast in bees’ (G), UVS birds’ (H), VS birds’ (I) and hummingbirds’ (J) vision. Phylogenetic regressions were performed using the function pgls in the package caper. The dashed line in (C) illustrates the theoretical discrimination threshold (1 JND) in the RNL model.

Phylogenetic regression analyses revealed that SP can affect floral conspicuousness significantly. For bees, there were positive correlations between floral chromatic contrast and SP intensity in both the CH (*R*^2^ = 0.270, *P* < 0.001; Fig. 6A) and the RNL models (*R*^2^ = 0.151, *P* < 0.001; Fig. 6C). Similarly, a positive correlation was found with respect to purity (*R*^2^ = 0.305, *P* < 0.001; Fig. 6B). For birds, chromatic contrast was inversely correlated with SP intensity (*R*^2^ = 0.156, *P* < 0.001; *R*^2^ < 0.122, *P* < 0.001; and *R*^2^ = 0.074, *P* = 0.001 in UVS birds’, VS birds’ and hummingbirds’ vision; Fig. 6D–F). In other words, a weak/absent SP reduced the colour conspicuousness of red flowers to bees, while simultaneously increasing the chromatic contrast to birds. For bees, the decreasing SP intensity reduced the chromatic contrast dramatically, from >1 JND (the theoretical discrimination threshold) to less than this value (Fig. 6C), while for birds, although chromatic contrast increased significantly along with the decrease in SP intensity, the absolute values were quite high (>10 JNDs generally).

## DISCUSSION

### Dual functional role of red floral colour

We investigated 130 species that produce red flowers. Compared with the other six colour categories, red is indeed (one of, at least) the most conspicuous colour to birds, and also the least conspicuous colour to bees, chromatically. The colour stimulates red photoreceptors in avian eyes strongly, while it stimulates other receptors weakly. Based on the colour opponency mechanism (which underlies the CH and RNL models), this extreme inequality of stimulation in different receptors should translate into strong colour perception of birds. In contrast, most bees have no red photoreceptors. Only green receptors were weakly excited by the red signals, making bees perceive true red as green. For birds, however, the absolute mean chromatic contrast values of all the floral colours we investigated were rather large (>5 JNDs; except white in VS birds’ vision model, 3.52 JNDs). This implied that the relative advantage of red coloration, although it occurs in perception estimation, may not necessarily contribute to higher attractiveness to the pollinators in the field, as any of the colours we tested are very likely to be detected readily (Lunau and Maier, 1995; Heystek *et al.*, 2014; Bergamo *et al.*, 2016). To bees, however, the absolute mean value of flower–background chromatic contrast of red flowers (1.07 JNDs) was quite small and close to the discrimination threshold of the corresponding model, meaning that it is difficult for bees to detect red flowers, especially on a complex leafy background (Rivest *et al.*, 2017; Telles *et al.*, 2017). In addition, the chromatic contrast was much lower than that of other floral colours. When flowers of other colours are available in the wild, red flowers will probably be an even more unlikely choice for bees, but will be more profitable for birds (in a resource partitioning scenario where bees and birds compete for nectar; Rodríguez-Gironés and Santamaría, 2004).

Achromatically, red is not the least stimulating colour to bees, suggesting the possibility that red can be detected by bees through an achromatic mechanism (Martínez-Harms *et al.*, 2010). However, this mechanism is more sensitive to light conditions and backgrounds and thus is a relatively unstable cue for pollinators (Kelber *et al.*, 2003). In fact, it has been suggested that the achromatic mechanism is mainly involved in detecting motion, edges (Lehrer *et al.*, 1990) and small targets (Giurfa *et al.*, 1997) rather than being involved in colour choice (Backhaus, 1991; Vorobyev and Brandt, 1997). Furthermore, it is more time-consuming for detecting colour stimuli than the chromatic mechanism (Giurfa *et al.*, 1996; Spaethe *et al.*, 2001). Therefore, the dual functional role of red floral colour in avoiding bees and attracting birds may be delivered mainly through the chromatic mechanism (Schaefer *et al.*, 2006).

### Shades of red between pollination systems and across continents

The differences in the shades of red flowers among pollination systems were subtle but significant. Generally, bee flowers, with higher chromatic contrast and purity, were more conspicuous to bees. Correspondingly, bird flowers performed better with respect to bird perception. Within bird pollination systems, the NW bird flowers, with higher chromatic contrast than OW bird flowers (either as a whole, or separately as bird flowers from Asia and Africa), may be more conspicuous to VS birds. Unexpectedly, these NW bird flowers may also be more attractive to UVS birds (e.g. sunbirds from the OW), despite the different colour vision between UVS and VS birds (Ödeen and Håstad, 2010).

Although many bird-pollinated red flowers are also visited by bees (Chittka and Waser, 1997), red flowers solely pollinated by bees are rare in nature. Although we made a particular effort to include such flowers in this study, only ten species were actually obtained (nine of them from the OW). Biochemically, it is not difficult to have an evolutionary transition from non-red (e.g. blue or purple, the typical bee flower colours) to red (Rausher, 2008; Tanaka *et al.*, 2008). Therefore, this rarity *per se* may suggest an evolutionary disadvantage for bee-pollinated flowers to be red.

### Influence of a secondary peak on colour conspicuousness

The differences in shades of red mentioned above may largely stem from the different properties of SPs in floral reflectance. Flowers pollinated by R− animals (e.g. bees) had higher SPs, possibly because an SP is an essential component for these animals to detect a signal through chromatic contrast and purity channels (Lunau *et al*., 1996, 2011). In contrast, SPs were lower in flowers pollinated by R+ animals, e.g. birds. To birds, SP reflectance decreases the chromatic contrast of the flower colour, possibly because the increased stimulation in avian SWS receptors (caused by the SP) counterbalances the stimulation in LWS receptors (excited by the primary reflectance at long wavelengths) through colour opponency mechanisms. A considerable proportion of red flowers that we collected are pollinated by butterflies. However, because not all flower-visiting butterflies have red photoreceptors, and their colour vision is not conservative enough even at the family level (Briscoe and Chittka, 2003), the SP pattern in butterfly flowers is not clear at present, but worth examining in the future. It is worth noting that those red flowers pollinated by beetles in this study all had low SPs (*k* range 0.014–0.146; mean ± s.e. 0.090 ± 0.025). Although most beetles lack red photoreceptors, the flower-visiting taxa are some of the exceptions (Martínez-Harms *et al.*, 2012), providing evidence to support this conclusion.

The conspicuousness difference between the two bird pollination systems (from the OW and the NW) can also be explained by much lower SP intensity in NW bird flowers. Given that red flowers with low SPs (possessing low SP intensity) perform better than flowers with high SPs (possessing high SP intensity) in avoiding bees (and possibly also in attracting birds) in terms of colour perception, this characteristic represents a more specialized floral colour phenotype in bird pollination systems. Therefore, the colours of bird flowers are more specialized in the NW than in the OW. This also coincides with the pattern found by Fleming and Muchhala (2008) whereby bird pollination systems are more specialized in the Neotropics than the Palaeotropics. Their conclusion was based on species richness data, while our study confirmed this pattern in a specific trait. This geographical pattern may be explained by the difference in flower–bird interaction between the OW and the NW (with a more specialized interaction in the NW; Zanata *et al.*, 2017), or by the difference in the diversity (higher in the NW) and spatiotemporal predictability (higher in the Neotropics) of floral resources between continents (Fleming and Muchhala, 2008). We assume that more intense competition between birds and bees or more frequent nectar robbing by bees (Rojas-Nossa *et al.*, 2016) in the NW may also act as a selection force and contribute to this pattern.

### The function and evolution of red floral colour

Being red endows flowers with the dual function of avoiding bees and attracting birds; being more specialized in red (with low SP) may result in more effective bee avoidance. There may be a fine transition from high-SP to low-SP red, towards a more specialized colour phenotype, which is likely to be biochemically derived. Anthocyanin, a common and widespread plant pigment, contributes to various floral colours from blue to red. It includes three major classes: pelargonidin-, cyanidin- and delphinidin-based anthocyanins (reviewed by Rausher, 2008), which alone (e.g. pelargonidin-based anthocyanin) or in combination (sometimes associated with carotenoids; Ng and Smith, 2016) may produce a red colour. A simple dehydroxylation process (loss-of-function mutation) enables the transformation from one anthocyanin class to another, gradually shifting colour from blue to redder colours (reviewed by Rausher, 2008). We noticed that although the NW flowers were dominated by low-SP red flowers, high-SP flowers still exist, suggesting the possibility of a gradual transition. Future studies based on a clear phylogenetic framework will be helpful to examine this shift.

Based on colour perception estimation and previous behavioural studies, we infer that better bee avoidance, rather than bird attraction, may have promoted this delicate shift. First, red flowers with high SP are conspicuous enough to birds (chromatic contrast >10 JNDs generally; Fig. 6D–F); the further increase in colour conspicuousness seems to be relatively unimportant. In contrast, this change could be crucial to bees, resulting in the chromatic contrast decrease from >1 JND to <1 JND (Fig. 6C). This may greatly impede target detection through the chromatic mechanism. Second, the perception difference between high SPs and low SPs to birds seems not to lead to differential behavioural responses (Meléndez-Ackerman *et al.*, 1997; Lunau *et al.*, 2011). Some birds even respond similarly between very different colours, e.g. white and pink, in the field (Bergamo *et al.*, 2016; but see Heystek *et al.*, 2014). For bees, however, it has been shown that the colour perception difference can successfully translate into different behavioural responses (Lunau *et al.*, 2011; Chen *et al.*, 2020).

To conclude, our results revealed the difference in shades of red between bee and bird flowers, and showed that such a difference may be common between flowers pollinated by R− and R+ animals. We also found a subtle but significant colour difference between red avian flowers from different continents. Unexpectedly, although UVS and VS birds differ in their colour vision, their visual selection with respect to shades of red may be similar. Low-SP red is a more effective colour than high-SP red for bee avoidance, representing a more specialized colour phenotype. And red flowers are more specialized in the NW than the OW. We inferred a delicate shift from high-SP to low-SP red in bird-pollinated flowers, which was more likely to have been driven by avoiding bees than attracting birds.

## SUPPLEMENTARY DATA

Supplementary data are available online at https://academic.oup.com/aob and consist of the following. Figure S1: phylogeny of 130 red flowers and the four traits of interest associated with them. Figure S2: phylogeny of 442 flowers and the colour associated with them. Table S1: list of 130 red flower species and relevant information. Methods S1: methods for calculating photon catch and chromatic contrast in the CH and RNL models.

mcaa103_suppl_Supplementary-Figures

mcaa103_suppl_Supplementary-Tables

mcaa103_suppl_Supplementary-Methods
