# Supplementary material for: Red flowers differ in shades between pollination systems and across continents
Source: Ann Bot. 2020 Jun 1;126(5):837–48. doi: 10.1093/aob/mcaa103 (PMC7539362; doi:10.1093/aob/mcaa103)
Supplement: mcaa103_suppl_Supplementary-Figures [file mcaa103_suppl_supplementary-figures.docx]

**Supplementary Figures**

***Annals of Botany***

**Original Article**

**Red flowers differ in shades between pollination systems and across continents**

Zhe Chen^1,2†^, Yang Niu^1*†^, Chang-Qiu Liu^3^ and Hang Sun^1*^

**
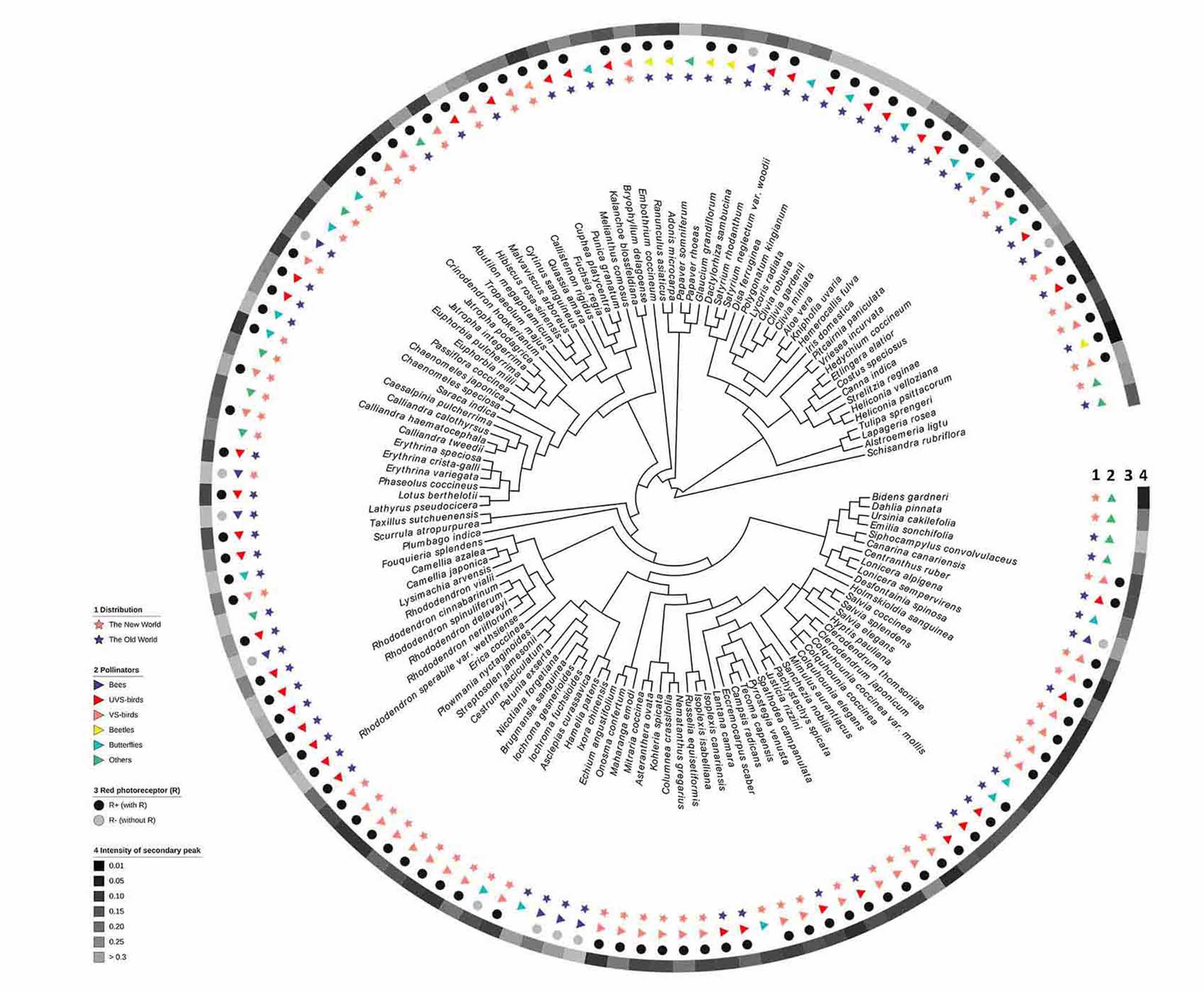
**

**Figure S1** Phylogeny of 130 red flower species and the four traits of interest associated with them. The phylogenetic tree was visualized using the online tool Evolview v2 (He *et al.*, 2016). Traits 1, 2, 3 and 4 are the distribution of the flowers, the pollinators of the flowers, the vision property (with and without red photoreceptor) of related pollinators, and the spectral characteristics (intensity of secondary peaks) of the flowers, respectively. Note that due to the uncertainty of the colour vision in certain pollinator groups, there are a few missing states for trait 3.


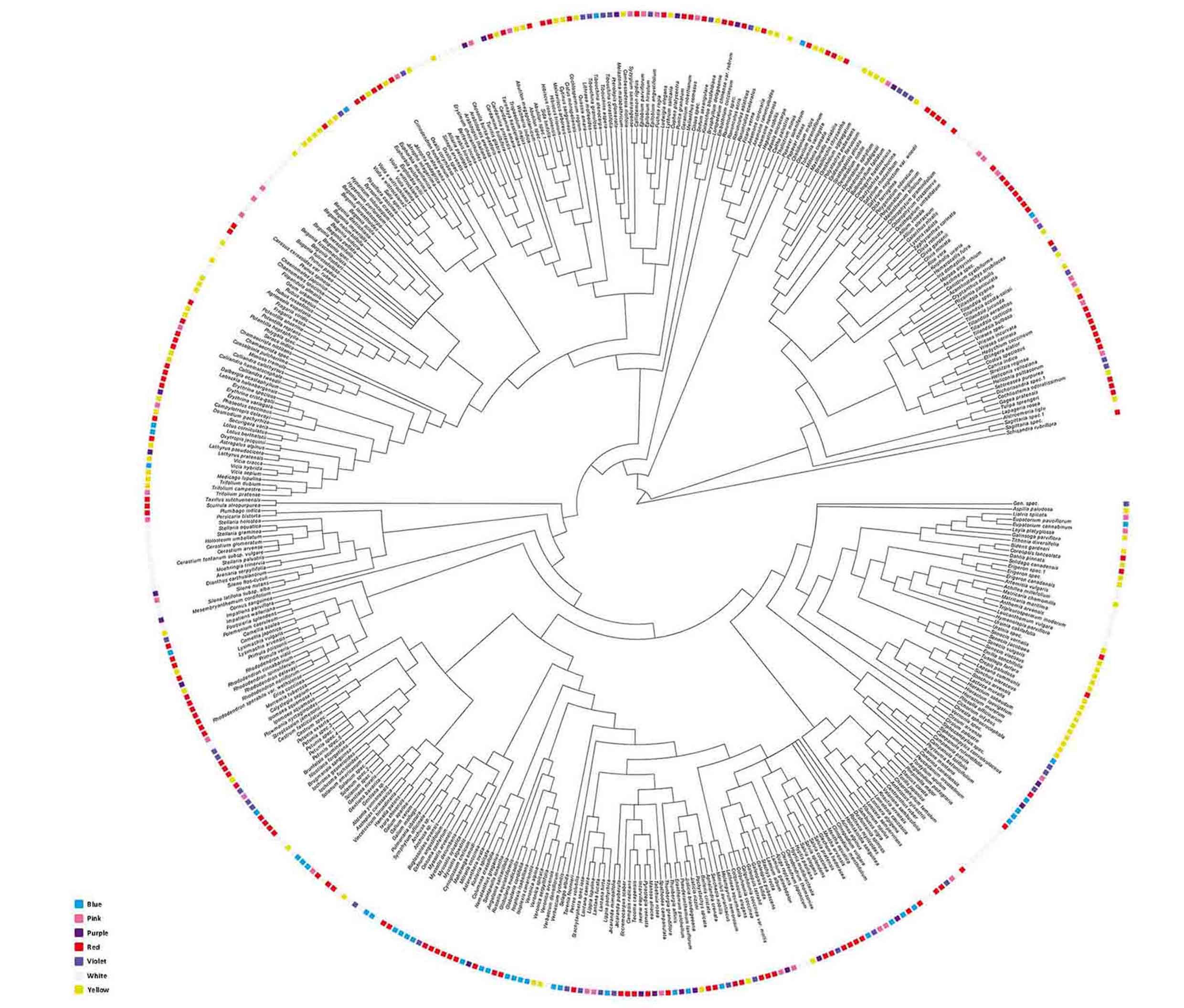


**Figure S2** Phylogeny of 442 flower species and the colour associated with them. The phylogenetic tree was visualized using the online tool Evolview v2 (He *et al.*, 2016). Flower colours are blue (*N* = 41), pink (*N* = 38), purple (*N* = 21), red (*N* =130), violet (*N* = 38), white (*N* = 86), yellow (*N* = 88).

**LITERATURE CITED**

**He Z-l, Zhang H-K, Gao S-H, Lercher MJ, Chen W-H, Hu S-N.** **2016**. Evolview v2: an online visualization and management tool for customized and annotated phylogenetic trees. *Nucleic Acids Research,* **44**: W236-W241.
