# Supplementary material for: Red flowers differ in shades between pollination systems and across continents
Source: Ann Bot. 2020 Jun 1;126(5):837–48. doi: 10.1093/aob/mcaa103 (PMC7539362; doi:10.1093/aob/mcaa103)
Supplement: mcaa103_suppl_Supplementary-Tables [file mcaa103_suppl_supplementary-tables.docx]

**Original Article**

**Red flowers differ in shades between pollination systems and across continents**

Zhe Chen^1,2†^, Yang Niu^1*†^, Chang-Qiu Liu^3^ and Hang Sun^1*^

**Table S1** List of 130 red flower species and relevant information

| **Species** | | **Native continents** | **SP intensity** | **Source of reflectance data** | **Pollinators** | **R** | **References for pollinators** |
| --- | --- | --- | --- | --- | --- | --- | --- |
| **Acanthaceae** | | | | | | | |
|  | *Justicia rizzini* | Americas | 0.067 | FReD | Hummingbirds | + | Buzato *et al.* (2000) (C); Schmidt-Lebuhn *et al.* (2007)(A) |
|  | *Pachystachys spicata* | Americas | 0.065 | XTBG | Hummingbirds | + | Schmidt-Lebuhn *et al.* (2007)(A) |
|  | *Sanchezia nobilis** | Americas | 0.191 | FReD | Hummingbirds | + | Willis (2002) (B); Mendonça and dos Anjos (2005) (C) |
| **Alstroemeriaceae** | | | | | | | |
|  | *Alstroemeria ligtu* | Americas | 0.216 | Botto-Mahan *et al.* (2011) | Insects | ? | Botto-Mahan *et al.* (2011) (C); González *et al.* (2014) (C); González *et al.* (2015) (C) |
| **Amaryllidaceae** | | | | | | | |
|  | *Clivia gardenii* | Africa | 0.422 | KBG | Birds (Passeriformes: Nectariniidae, sunbirds) | + | Kiepiel and Johnson (2014) (C) |
|  | *Clivia miniata* | Africa | 0.375 | KBG | Butterflies | + | Kiepiel and Johnson (2014) (C) |
|  | *Clivia robusta* | Africa | 0.455 | Kiepiel and Johnson (2014) | Birds (Passeriformes: Nectariniidae, sunbirds) | + | Kiepiel and Johnson (2014) (C) |
|  | *Lycoris radiata* | Asia | 0.442 | FReD | Butterflies | + | Observation |
| **Apocynaceae** | | | | | | | |
|  | *Asclepias curassavica** | Americas | 0.088 | FReD | Butterflies | - | Wyatt (1980) (B); Bierzychudek (1981) (B) |
| **Asparagaceae** | | | | | | | |
|  | *Polygonatum kingianum* | Asia | 0.379 | KBG | Birds | + | Prediction |
| **Asteraceae** | | | | | | | |
|  | *Bidens gardneri* | Americas | 0.040 | FReD | Insects | ? | Barbosa (1997) (C) |
|  | *Dahlia pinnata* | Americas | 0.163 | KBG | Insects | ? | Corrêa *et al.* (2001) (B); Bissa *et al.* (2011) (A) |
|  | *Emilia sonchifolia* | Asia | 0.192 | FReD | Insects | ? | Barbosa (1997) (C); Essien *et al.* (2013) (A) |
|  | *Ursinia cakilefolia* | Africa | 1.000 | FReD | Insects | ? | Goldblatt *et al.* (1998) (C); Ueckermann and Van Rooyen (2000) (C); de Waal *et al.* (2015) (C) |
| **Bignoniaceae** | | | | | | | |
|  | *Campsis radicans* | Americas | 0.090 | KBG | Hummingbirds | + | Bertin (1982) (C) |
|  | *Eccremocarpus scaber* | Americas | 0.062 | Martínez-Harms *et al.* (2010) | Hummingbirds | + | D'Arcy (1997) (A); Martínez-Harms *et al.* (2010) (B) |
|  | *Pyrostegia venusta* | Americas | 0.041 | KBG | Hummingbirds | + | Galetto *et al.* (1994) (B); Mostafa *et al.* (2013) (A) |
|  | *Spathodea campanulata* | Africa | 0.086 | XTBG | Birds (Passeriformes: Sturnidae, Corvidae (VS), & Dicruridae (VS)) | + | Rangaiah *et al.* (2004a) (B); Rangaiah and Solomon Raju (2017) (C) |
|  | *Tecoma capensis* | Africa | 0.075 | XTBG | Birds (Passeriformes: Nectariniidae & Zosteropidae) | + | Scott-Elliot (1890) (B); Nicolson (2002) (A); Galetto (2009) (A) |
| **Boraginaceae** | | | | | | | |
|  | *Echium angustifolium* | Europe & Asia & Africa | 0.152 | FReD | Bees | - | Dafni (1994) (B); Albaba (2015) (B) |
|  | *Maharanga emodi* | Asia | 0.317 | Xizang | Bees | - | Observation |
|  | *Onosma confertum* | Asia | 0.227 | SABT | Bees | - | Observation |
| **Bromeliaceae** | | | | | | | |
|  | *Pitcairnia paniculata* | Americas | 0.089 | FReD | Hummingbirds | + | Prediction; Krömer *et al.* (2006) (A) |
|  | *Vriesea incurvata** | Americas | 0.096 | FReD | Hummingbirds | + | de Queiroz Piacentini and Varassin (2007) (C); Silva and Piratelli (2014) (C) |
| **Campanulaceae** | | | | | | | |
|  | *Canarina canariensis* | Africa | 0.087 | Ollerton *et al.* (2009) | Birds (Passeriformes: Nectariniidae, sunbirds) | + | Ollerton *et al.* (2009) (C); Rodríguez-Rodríguez and Valido (2011) (C); Olesen *et al.* (2012) (C) |
|  | *Siphocampylus convolvulaceus* | Americas | 0.084 | FReD | Hummingbirds | + | Vizentin-Bugoni *et al.* (2014) (C) |
| **Cannaceae** | | | | | | | |
|  | *Canna indica* | Americas | 0.044 | XTBG | Hummingbirds | + | Glinos and Cocucci (2011) (C) |
| **Caprifoliaceae** | | | | | | | |
|  | *Centranthus ruber* | Europe & Asia & Africa | 0.172 | Kew | Butterflies | ? | Proctor *et al.* (1996) (A) |
|  | *Lonicera alpigena* | Europe | 0.239 | FReD | Bees | - | Robertson (1917) (C); Kato *et al.* (1993) (C) |
|  | *Lonicera sempervirens* | Americas | 0.113 | BBG | Hummingbirds | + | Miller and Nero (1983) (B); Hayden (2014) (A) |
| **Columelliaceae** | | | | | | | |
|  | *Desfontainia spinosa* | Americas | 0.038 | Martínez-Harms *et al.* (2010) | Hummingbirds | + | Smith-Ramirez (1993) (B); Martínez-Harms *et al.* (2010) (B) |
| **Costaceae** | | | | | | | |
|  | *Costus speciosus** | Asia & Oceania | 0.198 | XTBG | Bees | - | Sakai *et al.* 1999) (C); Jongjitvimol and Wattanachaiyingcharoen (2006) (B) |
| **Crassulaceae** | | | | | | | |
|  | *Bryophyllum delagoense* | Africa | 0.095 | KBG | Birds (Passeriformes: Nectariniidae, sunbirds) | + | Prediction |
|  | *Kalanchoe blossfeldiana* | Africa | 0.125 | KBG | Butterflies | ? | Observation |
| **Cytinaceae** | | | | | | | |
|  | *Cytinus sanguineus* | Africa | 0.312 | Hobbhahn and Johnson (2015) | Birds (Passeriformes: Nectariniidae, sunbirds) | + | Hobbhahn and Johnson (2015) (C) |
| **Elaeocarpaceae** | | | | | | | |
|  | *Crinodendron hookerianum* | Americas | 0.055 | Martínez-Harms *et al.* (2010) | Hummingbirds | + | Bricker (1992) (A); Aizen and Vázquez (2006) (A) |
| **Ericaceae** | | | | | | | |
|  | *Erica coccinea* | Africa | 0.134 | Malan (2013) | Birds (Passeriformes: Nectariniidae, sunbirds) | + | Malan (2013) (C); Heystek and Pauw (2014) (A) |
|  | *Rhododendron cinnabarinum* | Asia | 0.332 | Xizang | Birds | + | Prediction |
|  | *Rhododendron delavayi* | Asia | 0.088 | KBG | Birds (Passeriformes: Leiothrichidae: Heterophasia) | + | Huang *et al.* (2017) (C) |
|  | *Rhododendron neriiflorum* | Asia | 0.263 | Yunnan | Birds (Passeriformes: Nectariniidae & Phylloscopidae) | + | Huang *et al.* (2017) (C) |
|  | *Rhododendron sperabile var. wethsiense* | Asia | 0.305 | Yunnan | Birds (Passeriformes: Nectariniidae, sunbirds) | + | Observation |
|  | *Rhododendron spinuliferum* | Asia | 0.261 | KBG | Birds (Passeriformes: Nectariniidae, sunbirds) | + | Observation |
|  | *Rhododendron vialii* | Asia | 0.176 | KBG | Birds (Passeriformes: Nectariniidae, sunbirds) | + | Observation |
| **Euphorbiaceae** | | | | | | | |
|  | *Euphorbia milii* | Africa | 0.126 | XTBG | Bees | - | Taha *et al.* (2017) (B) |
|  | *Euphorbia pulcherrima** | Americas | 0.055 | FReD | Butterflies | ? | Haber *et al.* (1981) (A) |
|  | *Jatropha integerrima* | Americas | 0.159 | XTBG | Butterflies & Bees | ? | Gupta *et al.* (2007) (A); Gandhi and Kumar (2015) (B) |
|  | *Jatropha podagrica* | Americas | 0.062 | Kew | Butterflies | ? | Meerabai (2013) (A) |
| **Fabaceae** | | | | | | | |
|  | *Caesalpinia pulcherrima* | Americas | 0.065 | XTBG | Butterflies | + | Cruden and Hermann-Parker (1979) (C) |
|  | *Calliandra calothyrsus* | Americas | 0.164 | XTBG | Hawkmoths & Bats | ? | Hernáandez (1991) (A); Rajaselvam *et al.* (1995) (C); Chamberlain and Rajaselvam (1996) (C) |
|  | *Calliandra haematocephala* | Americas | 0.262 | XTBG | Hummingbirds | + | Nevling and Elias (1971) (A); Cruden *et al.* (1976) (A) |
|  | *Calliandra tweedii* | Americas | 0.121 | FReD | Hummingbirds & Bees | ? | Arroyo (1981) (?) |
|  | *Erythrina crista-galli* | Americas | 0.179 | KBG | Hummingbirds & Bees | ? | Galetto *et al.* (2000) (C); Costa and de Morais (2008) (C) |
|  | *Erythrina speciosa* | Americas | 0.121 | FReD | Hummingbirds | + | Vitali-Veiga and Machado (2000) (C); Almeida and Alves (2003) (C); Mendonça and dos Anjos (2006) (C) |
|  | *Erythrina variegata* | Europe & Asia & Africa & Oceania | 0.082 | Kew | Birds (Passeriformes: Nectariniidae, sunbirds) | + | Bruneau (1997) (A); Rangaiah *et al.* (2004b) (B); Solomon Raju *et al.* (2004) (B) |
|  | *Lathyrus pseudocicera* | Asia | 0.413 | FReD | Bees | - | Prediction |
|  | *Lotus berthelotii* | Africa | 0.072 | Ollerton *et al.* (2009) | Birds (Passeriformes: Phylloscopidae: Phylloscopus canariensis) | + | Ollerton *et al.* (2009) (C) |
|  | *Phaseolus coccineus* | Americas | 0.374 | Yunnan | Bees | - | Quagliotti and Marletto (1987) (C) |
|  | *Saraca indica* | Asia | 0.295 | XTBG | Butterflies | + | Observation |
| **Fouquieriaceae** | | | | | | | |
|  | *Fouquieria splendens* | Americas | 0.165 | Kew | Hummingbirds | + | Grant and Grant (1967) (C); Waser (1979) (C) |
| **Gesneriaceae** | | | | | | | |
|  | *Asteranthera ovata* | Americas | 0.160 | Martínez-Harms *et al.* (2010) | Hummingbirds | + | Smith-Ramírez *et al.* (2005) (C); Aizen and Vázquez (2006) (B) |
|  | *Columnea crassifolia* | Americas | 0.079 | Kew | Hummingbirds | + | Prediction |
|  | *Kohleria spicata* | Americas | 0.070 | Kew | Hummingbirds | + | Smith-Ramírez *et al.* (2005) (C); Martén‐Rodríguez *et al.* (2015) (C) |
|  | *Mitraria coccinea* | Americas | 0.049 | Martínez-Harms *et al.* (2010) | Hummingbirds | + | Smith-Ramírez *et al.* (2005) (C); Aizen and Rovere (2010) (C); Martínez-Harms *et al.* (2010) (B) |
|  | *Nematanthus gregarius* | Americas | 0.155 | Kew | Hummingbirds | + | Snow and Snow (1986) (C); Bergamo (2016) (C) |
| **Heliconiaceae** | | | | | | | |
|  | *Heliconia psittacorum* | Americas | 0.051 | XTBG | Hummingbirds | + | Feinsinger (1983) (C); Dobkin (1984) (B); da Cruzde *et al.* (2014) (C) |
|  | *Heliconia velloziana** | Americas | 0.089 | FReD | Hummingbirds | + | Snow and Teixeira (1982) (C); Sazima *et al.* (1995) (C) |
| **Iridaceae** | | | | | | | |
|  | *Iris domestica* | Asia | 0.324 | XTBG | Butterflies | + | Pickens (1936) (A); Kato (2000) (C) |
| **Lamiaceae** | | | | | | | |
|  | *Clerodendrum japonicum* | Asia | 0.116 | XTBG | Butterflies | + | Observation |
|  | *Clerodendrum thomsoniae* | Africa | 0.071 | XTBG | Butterflies | ? | Observation |
|  | *Colquhounia coccinea* | Asia | 0.086 | Xizang | Birds (Passeriformes: Nectariniidae, sunbirds) | + | Observation |
|  | *Colquhounia coccinea var. mollis* | Asia | 0.101 | KBG | Birds (Passeriformes: Nectariniidae, sunbirds) | + | Observation |
|  | *Colquhounia elegans* | Asia | 0.104 | KBG | Birds (Passeriformes: Nectariniidae, sunbirds) | + | Observation |
|  | *Holmskioldia sanguinea* | Asia | 0.082 | XTBG | Birds (Passeriformes: Nectariniidae, sunbirds) | + | Scogin (1988) (A) |
|  | *Hyptis pauliana* | Americas | 0.065 | FReD | Hummingbirds | + | Brantjes and de Vos (1981) (A) |
|  | *Salvia coccinea* | Americas | 0.040 | KBG | Hummingbirds | + | McGregor (1899) (A); Saito and Harborne (1992) (A) |
|  | *Salvia elegans* | Americas | 0.044 | KBG | Hummingbirds | + | Lara (2006) (C); Wester and Claßen-Bockhoff (2007) (B); Wester and Claßen-Bockhoff (2011) (A); Benitez-Vieyra *et al.* (2014) (B); Cuevas *et al.* (2018) (C) |
|  | *Salvia splendens* | Americas | 0.042 | KBG | Hummingbirds | + | Trelease (1881) (B); Wester and Claßen-Bockhoff (2007) (B); Wester and Claßen-Bockhoff (2011) (A) |
| **Liliaceae** | | | | | | | |
|  | *Tulipa sprengeri* | Europe & Asia & Africa | 0.014 | Kew | Beetles | + | Prediction |
| **Loranthaceae** | | | | | | | |
|  | *Scurrula atropurpurea* | Asia | 0.208 | KBG | Birds (Passeriformes: Dicaeidae & Nectariniidae) | + | Docters Van Leeuwen (1954) (B) |
|  | *Taxillus sutchuenensis* | Asia | 0.086 | Yunnan | Birds (Passeriformes: Nectariniidae, sunbirds) | + | Observation |
| **Lythraceae** | | | | | | | |
|  | *Cuphea platycentra** | Americas | 0.050 | XTBG | Hummingbirds | + | Prediction; Micheneau *et al.* (2006) (B) |
|  | *Punica granatum* | Europe & Asia & Africa | 0.101 | KBG | Birds | + | Pickens (1936) (B); Dhiman (2014) (C) |
| **Malvaceae** | | | | | | | |
|  | *Abutilon megapotamicum** | Americas | 0.088 | KBG | Hummingbirds | + | Gottsberger *et al.* (1984) (A); Andrade Lagos (2018) (B) |
|  | *Hibiscus rosa-sinensis* | Asia | 0.223 | FReD | Butterflies & Birds (Passeriformes, including sunbirds) | + | Prendergast (1982) (A); Subramanya and Radhamani (1993) (A); Zheng *et al.* (2013) (A) |
|  | *Malvaviscus arboreus* | Americas | 0.065 | FReD | Hummingbirds | + | Webb (1984) (B); Mendonça and dos Anjos (2005) (C); Villarreal (2016) (B) |
| **Melianthaceae** | | | | | | | |
|  | *Melianthus comosus* | Africa | 0.194 | KBG | Birds (Passeriformes: Zosteropidae, white-eyes) | + | Linder *et al.* (2006) (C) |
| **Myrtaceae** | | | | | | | |
|  | *Callistemon rigidus* | Oceania | 0.167 | KBG | Birds | + | Keighery and Perth (1979) (C); Corlett (2005) (B) |
| **Onagraceae** | | | | | | | |
|  | *Fuchsia regia** | Americas | 0.161 | FReD | Hummingbirds | + | Gottsberger *et al.* (1984) (A); Berry (1989) (A); Sazima *et al.* (1996) (C) |
| **Orchidaceae** | | | | | | | |
|  | *Dactylorhiza sambucina** | Europe | 0.165 | Gigord *et al.* (2002) | Bees | - | Jersáková *et al.* (2015) (B); Nilsson (1980) (C) |
|  | *Disa ferruginea* | Africa | 0.137 | de Jager *et al.* (2016) | Butterflies | ? | de Jager *et al.* (2016) (C); Johnson (1994) (C) |
|  | *Satyrium neglectum var. woodii* | Africa | 0.172 | Johnson and Van der Niet (2019) | Birds (Passeriformes: Nectariniidae, sunbirds) | + | Johnson and Van der Niet (2019) (C) |
|  | *Satyrium rhodanthum* | Africa | 0.111 | Van der Niet *et al.* (2015) | Birds (Passeriformes: Nectariniidae, sunbirds) | + | Van der Niet *et al.* (2015) (C) |
| **Papaveraceae** | | | | | | | |
|  | *Glaucium grandiflorum* | Europe & Asia & Africa | 0.146 | FReD | Beetles | + | Martínez-Harms *et al.* (2012) (B) |
|  | *Papaver rhoeas* | Europe & Asia & Africa | 0.146 | KBG | Beetles | + | Dafni *et al.* (1990) (B) |
|  | *Papaver somniferum* | Europe & Asia & Africa | 0.502 | FReD | Bees & Flies | ? | Miller *et al.* (2005) (C) |
| **Passifloraceae** | | | | | | | |
|  | *Passiflora coccinea* | Americas | 0.059 | XTBG | Hummingbirds | + | Storti (2002) (C); Fischer and Leal (2006) (C) |
| **Philesiaceae** | | | | | | | |
|  | *Lapageria rosea* | Americas | 0.240 | Martínez-Harms *et al.* (2010) | Hummingbirds | + | Smith-Ramírez *et al.* (2005) (C); Valdivia *et al.* (2006) (C) |
| **Phrymaceae** | | | | | | | |
|  | *Mimulus aurantiacus* | Americas | 0.034 | Martínez-Harms *et al.* (2010) | Hummingbirds | + | Streisfeld and Kohn (2007) (C); Belisle *et al.* (2012) (B); Handelman and Kohn (2014) (C) |
| **Plantaginaceae** | | | | | | | |
|  | *Isoplexis canariensis* | Africa | 0.124 | Ollerton *et al.* (2009) | Birds (Passeriformes: Phylloscopidae, Sylviidae, Fringillidae, & Paridae) | + | Valido *et al.* (2004) (C); Rodrãguez-Rodrãguez and Valido (2008) (C); Ollerton *et al.* (2009) (B) |
|  | *Isoplexis isabelliana* | Africa | 0.075 | Kew | Birds (Passeriformes: Phylloscopidae, Sylviidae, Fringillidae, & Paridae) | + | Valido *et al.* (2004) (C); Rodrãguez-Rodrãguez and Valido (2008) (B) |
|  | *Russelia equisetiformis* | Americas | 0.075 | XTBG | Hummingbirds | + | Prediction; Haynes *et al.* (2004) (B); Curtin and Fox (2014) (A) |
| **Plumbaginaceae** | | | | | | | |
|  | *Plumbago indica* | Asia | 0.328 | XTBG | Butterflies | + | Observation |
| **Primulaceae** | | | | | | | |
|  | *Lysimachia arvensis** | Europe & Asia & Africa | 0.606 | Ortiz *et al.* (2015) | Bees | - | Ortiz *et al.* (2015) (C) |
| **Proteaceae** | | | | | | | |
|  | *Embothrium coccineum* | Americas | 0.185 | Martínez-Harms *et al.* (2010) | Hummingbirds | + | Smith-Ramirez and Armesto (2010) (C); Chalcoff *et al.* (2012) (C) |
| **Ranunculaceae** | | | | | | | |
|  | *Adonis microcarpa* | Europe & Asia | 0.066 | FReD | Beetles | + | Martínez-Harms *et al.* (2012) (C) |
|  | *Ranunculus asiaticus* | Europe & Asia & Africa | 0.078 | FReD | Beetles | + | Dafni *et al.* (1990) (C); Dafni and Potts (2004) (B); Martínez-Harms *et al.* (2012) (B) |
| **Rosaceae** | | | | | | | |
|  | *Chaenomeles japonica* | Asia | 0.188 | KBG | Birds (Passeriformes: Nectariniidae, sunbirds) | + | Observation |
|  | *Chaenomeles speciosa* | Asia | 0.202 | KBG | Birds (Passeriformes: Nectariniidae, sunbirds) | + | Observation; Fang *et al.* (2012) (B) |
| **Rubiaceae** | | | | | | | |
|  | *Hamelia patens* | Americas | 0.063 | XTBG | Hummingbirds | + | Lasso and Naranjo (2003) (C) |
|  | *Ixora chinensis* | Asia | 0.236 | XTBG | Butterflies | ? | Duara (2014) (C); Akter *et al.* (2015) (B) |
| **Schisandraceae** | | | | | | | |
|  | *Schisandra rubriflora* | Asia | 0.108 | Kew | Insects | ? | Luo *et al.* (2018) (B) |
| **Simaroubaceae** | | | | | | | |
|  | *Quassia amara* | Americas | 0.226 | Kew | Hummingbirds | + | Baker (1975) (A); Roubik *et al.* (1985) (C) |
| **Solanaceae** | | | | | | | |
|  | *Brugmansia sanguinea* | Americas | 0.057 | Ng and Smith (2016) | Hummingbirds | + | Velasco Parreño (2016) (A) |
|  | *Cestrum fasciculatum* | Americas | 0.049 | Ng and Smith (2016) | Hummingbirds | + | Prediction |
|  | *Iochroma fuchsioides* | Americas | 0.153 | Ng and Smith (2016) | Hummingbirds | + | Smith *et al.* (2008) (C) |
|  | *Iochroma gesnerioides* | Americas | 0.051 | Ng and Smith (2016) | Hummingbirds | + | Smith *et al.* (2008) (C) |
|  | *Nicotiana forgetiana* | Americas | 0.096 | Ng and Smith (2016) | Hummingbirds | + | Ippolito *et al.* (2004) (C); Kaczorowski *et al.* (2005) (C) |
|  | *Petunia exserta* | Americas | 0.126 | Ng and Smith (2016) | Hummingbirds | + | Stehmann (1987) (A); Lorenz-Lemke *et al.* (2006) (B) |
|  | *Plowmania nyctaginoides* | Americas | 0.076 | Ng and Smith (2016) | Hummingbirds | + | Knapp (2010) (A) |
|  | *Streptosolen jamesonii* | Americas | 0.051 | Ng and Smith (2016) | Hummingbirds | + | Cocucci (1995) (A) |
| **Strelitziaceae** | | | | | | | |
|  | *Strelitzia reginae** | Africa | 0.040 | Yunnan | Birds (Passeriformes: Nectariniidae, sunbirds) | + | Frost and Frost (1981) (B); Coombs and Peter (2009) (B) |
| **Theaceae** | | | | | | | |
|  | *Camellia azalea* | Asia | 0.357 | KBG | Bees & Butterflies | ? | Luo *et al.* (2011) (C) |
|  | *Camellia japonica* | Asia | 0.212 | Kew | Birds (Passeriformes: Nectariniidae, sunbirds) | + | Yumoto (1987) (C); Kunitake *et al.* (2004) (C) |
| **Tropaeolaceae** | | | | | | | |
|  | *Tropaeolum majus* | Americas | 0.042 | KBG | Hummingbirds | + | Baker (1975) (A) |
| **Verbenaceae** | | | | | | | |
|  | *Lantana camara* | Americas | 0.082 | XTBG | Butterflies | ? | Barrows (1976) (A); Schemske (1976) (C) |
| **Xanthorrhoeaceae** | | | | | | | |
|  | *Aloe vera* | Africa | 0.197 | KBG | Birds (Passeriformes: Nectariniidae, sunbirds) | + | Ash *et al.* (1961) (B); Ghadirian *et al.* (2007) (C); Jain *et al.* (2013) (C) |
|  | *Hemerocallis fulva* | Asia | 0.227 | KBG | Butterflies | + | Hirota *et al.* (2012) (C); Hirota *et al.* (2013) (C) |
|  | *Kniphofia uvaria* | Africa | 0.112 | de Jager *et al.* (2016) | Birds | + | Pickens (1936) (A); Johnson (1994) (B) |
| **Zingiberaceae** | | | | | | | |
|  | *Etlingera elatior* | Asia | 0.232 | XTBG | Birds (Passeriformes: Nectariniidae, spiderhunter) | + | Classen (1987) (B); Sakai *et al.* (2013) (C) |
|  | *Hedychium coccineum* | Asia | 0.187 | KBG | Butterflies | + | Kittipanangkul and Ngamriabsakul (2006) (A); Gao *et al.* (2012) (C) |

Note: A species with an asterisk * indicates the measurement of floral accessories (bract or calyx), or the major red part of the flower, or the red morph where different colour morphs exist. In the column “Source of reflectance data”, “BBG”, “KBG”, “SABG” and “XTBG” are the abbreviations for “the Beijing Botanical Garden”, “Kunming Botanical Garden”, “Shangri-La Alpine Botanical Garden” and “Xishuangbanna Tropical Botanical Garden” respectively; “FReD” refers to the Floral Reflectance Database (FReD, http://www.reflectance.co.uk, Arnold *et al.*, 2008). “SP” refers to the secondary peak of the floral reflectance. “R” refers to the red photoreceptor of corresponding pollinators, “-” and “+” represent absent and present respectively, whilst “?” represents uncertainty about the state of the red photoreceptors. Bees are R- animals (Peitsch *et al.*, 1992). Avian pollinators (both in the Old and New World) are R+ animals (Hart and Hunt, 2007; Cronk and Ojeda, 2008). Butterflies have relatively complex characteristics (Briscoe and Chittka, 2003). “Prediction” in the last column means that pollinators of the corresponding plant were predicted based on several floral reproductive traits. The letters in parentheses after the references indicate that the pollinators are determined either without providing detailed evidence (which may be based on reviews of previous research or pollination syndrome) (A), or determined by qualitative (B) or quantitative (C) methods on frequency/proportion of visits, visitation rate, pollinator efficiency, seed/fruit set, pollen load/transfer or contact with stigma.

**LITERATURE CITED**

**Aizen MA, Rovere AE.** **2010**. Reproductive interactions mediated by flowering overlap in a temperate hummingbird-plant assemblage. *Oikos,* **119**: 696-706.

**Aizen MA, Vázquez DP.** **2006**. Flowering phenologies of hummingbird plants from the temperate forest of southern South America: is there evidence of competitive displacement? *Ecography,* **29**: 357-366.

**Akter F, Habib MSA, Hossain MM.** **2015**. Spatial and temporal dimensions of butterfly species diversity in Jahangirnagar University campus and its suburbs, Bangladesh. *Jahangirnagar University Journal of Biological Sciences,* **4**: 27-40.

**Albaba I.** **2015**. A list of important honeybee nectariferous and polleniferous plant species in the West Bank Governorates, Palestine. *Journal of Agricultural Science and Technology,* **5**: 114-121.

**Almeida EM, Alves MAS.** **2003**. Comportamento de Aves nectarívoras em *Erythrina speciosa* Andrews (Leguminosae-Papilionoideae) em uma área de Floresta Atlântica, Ilha Grande, Rio de Janeiro. *Revista de Etologia,* **5**: 15-21.

**Andrade Lagos ML.** **2018**. *Estudio preliminar de las plantas que usan las Aves nectarívoras para su alimentación en tres áreas verdes de Bogotá,* MSc Thesis, Universidad Distrital Francisco José de Caldas, Bogotá, Colombia.

**Arnold SEJ, Savolainen V, Chittka L.** **2008**. FReD: the floral reflectance spectra database. *Nature Precedings*. doi: 10.1038/npre.2008.1846.1

**Arroyo MTK.** **1981.** Breeding systems and pollination biology in Leguminosae. In: Polhill RM, Raven PH, eds. *Advances in Legume systematics*. Kew, UK: Royal Botanic Gardens.

**Ash JS, Jones PH, Melville R.** **1961**. The contamination of birds with pollen and other substances. *British Birds,* **54**: 93-100.

**Baker HG.** **1975**. Sugar concentrations in nectars from hummingbird flowers. *Biotropica,* **7**: 37-41.

**Barbosa AAA.** **1997**. *Biologia reprodutiva de uma comunidade de Campo Sujo, Uberlandia/MG,* PhD Thesis, Universidade Estadual de Campinas, Campinas.

**Barrows EM.** **1976**. Nectar robbing and pollination of *Lantana camara* (Verbenaceae). *Biotropica,* **8**: 132-135.

**Belisle M, Peay KG, Fukami T.** **2012**. Flowers as islands: spatial distribution of nectar-inhabiting microfungi among plants of *Mimulus aurantiacus*, a hummingbird-pollinated shrub. *Microbial Ecology,* **63**: 711-718.

**Benitez-Vieyra S, Fornoni J, Pérez-Alquicira J, Boege K, Domínguez CA.** **2014**. The evolution of signal-reward correlations in bee- and hummingbird-pollinated species of *Salvia*. *Proceedings of the Royal Society of London B,* **281**: 20132934.

**Bergamo PJ.** **2016**. *Community assembly and potential for indirect effects of hummingbird-pollinated plants in the Atlantic forest,* MSc Thesis, Universidade Estadual de Campinas, Campinas, Brazil.

**Berry PE.** **1989**. A systematic revision of *Fuchsia* Sect. *Quelusia* (Onagraceae). *Annals of the Missouri Botanical Garden,* **76**: 532-584.

**Bertin RI.** **1982**. Floral biology, hummingbird pollination and fruit production of trumpet creeper (*Campsis radicans*, Bignoniaceae). *American Journal of Botany,* **69**: 122-134.

**Bierzychudek P.** **1981**. *Asclepias*, *Lantana*, and *Epidendrum*: a floral mimicry complex? *Biotropica,* **13**: 54-58.

**Bissa S, Bohra A, Bohra A.** **2011**. Screening of *Dahlia pinnata* for its antimicrobial activity. *Journal of Research in Biology,* **1**: 51-55.

**Botto-Mahan C, Ramírez PA, Ossa CG, Medel R, Ojeda-Camacho M, González AV.** **2011**. Floral herbivory affects female reproductive success and pollinator visitation in the perennial herb *Alstroemeria ligtu* (Alstroemeriaceae). *International Journal of Plant Sciences,* **172**: 1130-1136.

**Brantjes NBM, de Vos OC.** **1981**. The explosive release of pollen in flowers of *Hyptis* (Lamiaceae). *New Phytologist,* **87**: 425-430.

**Bricker JS.** **1992**. Pollination biology of the genus *Crinodendron* (Elaeocarpaceae). *Journal of the Arizona-Nevada Academy of Science,* **24/25**: 51-54.

**Briscoe AD, Chittka L.** **2003**. The evolution of color vision in insects. *Annual Review of Entomology,* **46**: 471-510.

**Bruneau A.** **1997**. Evolution and homology of bird pollination syndromes in *Erythrina* (Leguminosae). *American Journal of Botany,* **84**: 54-71.

**Buzato S, Sazima M, Sazima I.** **2000**. Hummingbird-pollinated floras at three Atlantic forest sites. *Biotropica,* **32**: 824-841.

**Chalcoff VR, Aizen MA, Ezcurra C.** **2012**. Erosion of a pollination mutualism along an environmental gradient in a south Andean treelet, *Embothrium coccineum* (Proteaceae). *Oikos,* **121**: 471-480.

**Chamberlain JR, Rajaselvam RJ.** **1996.** *Calliandra calothyrsus* pollinator behaviour and seed production. In: Evans DO, ed. *Proceeding international workshop on the genus Calliandra*. Morrilton, US: Winrock International Institute for Agriculture Development.

**Classen R.** **1987**. Morphological adaptations for bird pollination in *Nicolaia elatior* (Jack) Horan (Zingiberaceae). *Gardens' Bulletin (Singapore),* **40**: 37-43.

**Cocucci A.** **1995**. Floral mechanisms in the Tribe Salpiglossidae (Solanaceae). *Plant Systematics and Evolution,* **194**: 207-230.

**Coombs G, Peter CI.** **2009**. Do floral traits of *Strelitzia reginae* limit nectar theft by sunbirds? *South African Journal of Botany,* **75**: 751-756.

**Corlett RT.** **2005**. Interactions between birds, fruit bats and exotic plants in urban Hong Kong, South China. *Urban Ecosystems,* **8**: 275-283.

**Corrêa CA, Irgang BE, Moreira GRP.** **2001**. Estrutura floral das angiospermas usadas por *Heliconius erato phyllis* (Lepidoptera, Nymphalidae) no Rio Grande do Sul, Brasil. *Iheringia Série Zoologia,* **22**: 71-84.

**Costa RACV, de Morais ABB.** **2008**. Fenologia e visitantes florais de *Erythrina crista-galli* L. (Leguminosae: Faboideae) em Santa Maria, Rio Grande do Sul, Brasil. *Biotemas,* **21**: 51-56.

**Cronk Q, Ojeda I.** **2008**. Bird-pollinated flowers in an evolutionary and molecular context. *Journal of Experimental Biology,* **59**: 715-727.

**Cruden RW, Hermann-Parker SM.** **1979**. Butterfly pollination of *Caesalpinia pulcherrima*, with observations on a psychophilous syndrome. *Journal of Ecology,* **67**: 155-168.

**Cruden RW, Kinsman S, Stockhouse RE, II, Linhart YB.** **1976**. Pollination, fecundity, and the distribution of moth-flowered plants. *Biotropica,* **8**: 204-210.

**Cuevas E, Espino J, Marques I.** **2018**. Reproductive isolation between *Salvia elegans* and *S. fulgens*, two hummingbird-pollinated sympatric sages. *Plant Biology,* **20**: 1075-1082.

**Curtin S, Fox D.** **2014.** Human dimensions of wildlife gardening: its development, controversies and psychological benefits. In: Dixon G, Aldous DE, eds. *Horticulture: Plants for people and places*. Heidelberg, Germany: Springer.

**D'Arcy WG.** **1997**. A review of the genus *Eccremocarpus* (Bignoniaceae). *Annals of the Missouri Botanical Garden,* **84**: 103-111.

**da Cruz DD, de Castro e Silva M, de Albuquerque NSL.** **2014**. Floral syndrome, resources and pollinator guilds: A case study of *Heliconia psittacorum* L. f. (Heliconiaceae). *Iheringia Serie Botanica,* **69**: 303-312.

**Dafni A.** **1994**. Note on side advertisement in flowers. *Functional Ecology,* **8**: 136-138.

**Dafni A, Bernhardt P, Shmida A*, et al.*** **1990**. Red bowl-shaped flowers: convergence for beetle pollination in the Mediterranean region. *Israel Journal of Botany,* **39**: 81-92.

**Dafni A, Potts SG.** **2004**. The role of flower inclination, depth, and height in the preferences of a pollinating beetle (Coleoptera: Glaphyridae). *Journal of Insect Behavior,* **17**: 823-834.

**de Jager M, Newman E, Theron G, Botha P, Barton M, Anderson B.** **2016**. Pollinators can prefer rewarding models to mimics: consequences for the assumptions of Batesian floral mimicry. *Plant Systematics and Evolution,* **302**: 409-418.

**de Queiroz Piacentini V, Varassin IG.** **2007**. Interaction network and the relationships between bromeliads and hummingbirds in an area of secondary Atlantic rain forest in southern Brazil. *Journal of Tropical Ecology,* **23**: 663-671.

**de Waal C, Anderson B, Ellis AG.** **2015**. Relative density and dispersion pattern of two southern African Asteraceae affect fecundity through heterospecific interference and mate availability, not pollinator visitation rate. *Journal of Ecology,* **103**: 513-525.

**Dhiman J.** **2014**. *Studies on reproductive biology of wild pomegranate (Punica granatum L.),* MSc Thesis, College of Forestry, Solan, India.

**Dobkin DS.** **1984**. Flowering patterns of long-lived *Heliconia* inflorescences: implications for visiting and resident nectarivores. *Oecologia,* **64**: 245-254.

**Docters van Leeuwen WM.** **1954**. On the biology of some Javanese Loranthaceae and the role birds play in their life-historie. *Beaufortia,* **4**: 103-207.

**Duara P.** **2014**. Effectiveness and importance of butterflies as pollinators to the flowers of *Ixora coccinea*. *International Journal of Research Studies in Biosciences,* **2**: 71-74.

**Essien GE, Nwidu LL, Nwafor PA.** **2013**. Anti-inflammatory and analgesic potential of methanolic extract of *Emilia sonchifolia* (Compositae) leaves in rodents. *African Journal Biomedical Research,* **12**: 199-207.

**Fang Q, Chen YZ, Huang SQ.** **2012**. Generalist passerine pollination of a winter-flowering fruit tree in central China. *Annals of Botany,* **109**: 379-384.

**Feinsinger P.** **1983**. Variable nectar secretion in a *Heliconia* species pollinated by hermit hummingbirds. *Biotropica,* **15**: 48-52.

**Fischer E, Leal IR.** **2006**. Effect of nectar secretion rate on pollination success of *Passiflora coccinea* (Passifloraceae) in the Central Amazon. *Brazilian Journal of Biology,* **66**: 747-754.

**Frost SK, Frost PGH.** **1981**. Sunbird pollination of *Strelitzia nicolai*. *Oecologia,* **49**: 379-384.

**Galetto L.** **2009**. Nectary and nectar features: occurrence, significance, and trends in Bignoniaceae. *The Journal of Plant Reproductive Biology,* **1**: 121-132.

**Galetto L, Bernardello G, Isele IC, Vesprini J, Speroni G, Berduc A.** **2000**. Reproductive biology of *Erythrina crista-galli* (Fabaceae). *Annals of the Missouri Botanical Garden,* **87**: 127-145.

**Galetto L, Bernardello LM, Juliani HR.** **1994**. Characteristics of secretion of nectar in *Pyrostegia venusta* (Ker-Gawl.) Miers (Bignoniaceae). *New Phytologist,* **127**: 465-471.

**Gandhi S, Kumar D.** **2015**. Studies on butterfly diversity, abundance and utilization of plant resources in urban localities of Banyan city-Vadodara, Gujarat, India. *Journal of Entomology and Zoology Studies,* **3**: 476-480.

**Gao JY, Sheng CL, Yang SX.** **2012**. Adaptive significance of mass-flowering in *Hedychium coccineum* (Zingiberaceae). *Biodiversity Science,* **20**: 376-385.

**Ghadirian T, Qashqaei AT, Dadras M.** **2007**. Notes on feeding and breeding habits of the purple sunbird *Nectarinia asiatica* (*Cinnyris asiaticus*) in Bandar Abbas, Hormozgan, Southern Iran. *Podoces,* **2**: 122-126.

**Gigord LDB, Macnair MR, Stritesky M, Smithson A.** **2002**. The potential for floral mimicry in rewardless orchids: an experimental study. *Proceedings of the Royal Society of London B,* **269**: 1389-1395.

**Glinos E, Cocucci AA.** **2011**. Pollination biology of *Canna indica* (Cannaceae) with particular reference to the functional morphology of the style. *Plant Systematics and Evolution,* **291**: 49-58.

**Goldblatt P, Bernhardt P, Manning JC.** **1998**. Pollination of petaloid geophytes by monkey beetles (Scarabaeidae: Rutelinae: *Hopliini*) in southern Africa. *Annals of the Missouri Botanical Garden,* **85**: 215-230.

**González AV, Murúa M, Ramírez PA.** **2014**. Temporal and spatial variation of the pollinator assemblages in *Alstroemeria ligtu* (Alstroemeriaceae). *Revista Chilena de Historia Natural,* **87**: 1-4.

**González AV, Murúa MM, Pérez F.** **2015**. Floral integration and pollinator diversity in the generalized plant-pollinator system of *Alstroemeria ligtu* (Alstroemeriaceae). *Evolutionary Ecology,* **29**: 63-75.

**Gottsberger G, Schrauwen J, Linskens HF.** **1984**. Amino acids and sugars in nectar, and their putative evolutionary significance. *Plant Systematics and Evolution,* **145**: 55-77.

**Grant KA, Grant V.** **1967**. Effects of hummingbird migration on plant speciation in the California flora. *Evolution,* **21**: 457-465.

**Gupta G, Chauhan S, Chauhan SVS.** **2007.** Reproductive biology of *Jatropha integerrima* Jacq. In: Solomon Raju AJ, ed. *Advances in pollen spore research, Volume XXIV*. New Delhi, India: Today and Tomorrow's Printers & Publishers.

**Haber WA, Frankie GW, Baker HG, Baker I, Koptur S.** **1981**. Ants like flower nectar. *Biotropica,* **13**: 211-214.

**Handelman C, Kohn JR.** **2014**. Hummingbird color preference within a natural hybrid population of *Mimulus aurantiacus* (Phrymaceae). *Plant Species Biology,* **29**: 65-72.

**Hart NS, Hunt DM.** **2007**. Avian visual pigments: characteristics, spectral tuning, and evolution. *The American Naturalist,* **169**: S7-S26.

**Hayden WJ.** **2014**. *2014 Virginia wildflower of the year: coral honeysuckle, Lonicera sempervirens*: Virginia Native Plant Society.

**Haynes J, Mclaughlin J, Vasquez L, Hunsberger A.** **2004**. Low-maintenance landscape plants for south Florida. *Environmental Horticulture Department., Florida Cooperative Extension Service, University of Florida-IFAS Publication ENH854, Florida, USA.*

**Hernáandez HM.** **1991**. Taxonomy, geographical distribution and reproductive biology of *Calliandra calothyrsus* (Leguminosae, Mimosoideae), a species with agroforestry potential. *Anales del Instituto de Biología. Serie Botánica,* **62**: 121-131.

**Heystek A, Pauw A.** **2014**. Does competition for pollinators contribute to structuring *Erica* communities? *Journal of Vegetation Science,* **25**: 648-656.

**Hirota SK, Nitta K, Kim Y*, et al.*** **2012**. Relative role of flower color and scent on pollinator attraction: experimental tests using F1 and F2 hybrids of daylily and nightlily. *Plos One,* **7**: e39010. doi: 10.1371/journal.pone.0039010

**Hirota SK, Nitta K, Suyama Y, Kawakubo N, Yasumoto AA, Yahara T.** **2013**. Pollinator-mediated selection on flower color, flower scent and flower morphology of *hemerocallis*: evidence from genotyping individual pollen grains on the stigma. *PloS One,* **10**: e0117885. doi: 10.1371/journal.pone.0085601

**Hobbhahn N, Johnson SD.** **2015**. Sunbird pollination of the dioecious root parasite *Cytinus sanguineus* (Cytinaceae). *South African Journal of Botany,* **99**: 138-143.

**Huang ZH, Song YP, Huang SQ.** **2017**. Evidence for passerine bird pollination in *Rhododendron* species. *AoB Plants,* **9**: plx062. doi: 10.1093/aobpla/plx062

**Ippolito A, Fernes GW, Holtsford TP.** **2004**. Pollinator preferences for *Nicotiana alata*, *N. forgetiana*, and their F1 hybrids. *Evolution,* **58**: 2634-2644.

**Jain AK, Sharma BK, Bhat AA.** **2013**. Flowering phenology and floral visitors of some medicinal plants of Gwalior, Madhya Pradesh, India. *The International Journal of Plant Reproductive Biology,* **5**: 81-84.

**Jersáková J, Traxmandlová I, Ipser Z*, et al.*** **2015**. Biological flora of Central Europe: *Dactylorhiza sambucina* (L.) Soó. *Perspectives in Plant Ecology Evolution and Systematics,* **17**: 318-329.

**Johnson SD.** **1994**. Evidence for Batesian mimicry in a butterfly‐pollinated orchid. *Biological Journal of the Linnean Society,* **53**: 91-104.

**Johnson SD, Van der Niet T.** **2019**. Bird pollination in an African *Satyrium* (Orchidaceae) confirmed by camera traps and selective exclusion experiments. *Plant Systematics and Evolution,* **305**: 477-484.

**Jongjitvimol T, Wattanachaiyingcharoen W.** **2006**. Pollen food sources of the stingless bees *Trigona apicalis* Smith, 1857, *Trigona collina* Smith, 1857 and *Trigona fimbriata* Smith, 1857 (Apidae, Meliponinae) in Thailand. *The Natural History Journal of Chulalongkorn University,* **6**: 75-82.

**Kaczorowski RL, Gardener MC, Holtsford TP.** **2005**. Nectar traits in *Nicotiana* section *Alatae* (Solanaceae) in relation to floral traits, pollinators, and mating system. *American Journal of Botany,* **92**: 1270-1283.

**Kato M.** **2000**. Anthophilous insect community and plant-pollinator interactions on Amami Islands in the Ryukyu Archipelago, Japan. *Contributions from the Biological Laboratory, Kyoto University,* **29**: 157-254.

**Kato M, Matsumoto M, Kato T.** **1993**. Flowering phenology and anthophilous insect community in the cool-temperate subalpine forests and meadows at Mt. Kushigata in the central part of Japan. *Contributions from the Biological Laboratory Kyoto University,* **28**: 119-172.

**Keighery GJ, Perth W.** **1979**. Bird pollination in south western Australia: a checklist. *Plant Systematics and Evolution,* **135**: 171-176.

**Kiepiel I, Johnson SD.** **2014**. Shift from bird to butterfly pollination in *Clivi* (Amaryllidaceae). *American Journal of Botany,* **101**: 190-200.

**Kittipanangkul N, Ngamriabsakul C.** **2006**. Pollen and pollinator limitation of seed initiation in *Etlingera littoralis* (J. K nig) Giseke (Zingiberaceae) in Klong Klai Basin, Khao Nan National Park, Thailand. *Walailak Journal of Science and Technology,* **3**: 207-217.

**Knapp S.** **2010**. On ‘various contrivances’: pollination, phylogeny and flower form in the Solanaceae. *Philosophical Transactions of the Royal Society B,* **365**: 449-460.

**Krömer T, Kessler M, Herzog SK.** **2006**. Distribution and flowering ecology of bromeliads along two climatically contrasting elevational transects in the Bolivian Andes. *Biotropica,* **38**: 183–195.

**Kunitake YK, Hasegawa M, Miyashita T, Higuchi H.** **2004**. Role of a seasonally specialist bird *Zosterops japonica* on pollen transfer and reproductive success of *Camellia japonica* in a temperate area. *Plant Species Biology,* **19**: 197-201.

**Lara C.** **2006**. Temporal dynamics of flower use by hummingbirds in a highland temperate forest in Mexico. *Ecoscience,* **13**: 23-29.

**Lasso E, Naranjo ME.** **2003**. Effect of pollinators and nectar robbers on nectar poroduction and pollen deposition in *Hamelia patens* (Rubiaceae). *Biotropica,* **35**: 57-66.

**Linder HP, Dlamini T, Henning J, Verboom GA.** **2006**. The evolutionary history of *Melianthus* (Melianthaceae). *American Journal of Botany,* **93**: 1052-1064.

**Lorenz-Lemke AP, Mäder G, Muschner VC*, et al.*** **2006**. Diversity and natural hybridization in a highly endemic species of *Petunia* (Solanaceae): a molecular and ecological analysis. *Molecular Ecology,* **15**: 4487-4497.

**Luo SX, Zhang LJ, Yuan S, Ma ZH, Zhang DX, Renner SS.** **2018**. The largest early-diverging angiosperm family is mostly pollinated by ovipositing insects and so are most surviving lineages of early angiosperms. *Proceedings of the Royal Society of London B,* **285**: 20172365.

**Luo XY, Tang GD, Mo LJ, Zhuang XY.** **2011**. Pollination biology of *Camellia changii*. *Chinese Journal of Ecology,* **30**: 552-557.

**Malan M.** **2013**. *Intraspecific variation in Erica coccinea,* MSc Thesis, University of Cape Town, South Africa.

**Martén‐Rodríguez S, Quesada M, Castro AA, Lopezaraiza‐Mikel M, Fenster CB.** **2015**. A comparison of reproductive strategies between island and mainland Caribbean Gesneriaceae. *Journal of Ecology,* **103**: 1190-1204.

**Martínez-Harms J, Palacios AG, Márquez N, Estay P, Arroyo MTK, Mpodozis J.** **2010**. Can red flowers be conspicuous to bees? *Bombus dahlbomii* and South American temperate forest flowers as a case in point. *Journal of Experimental Biology,* **213**: 564-571.

**Martínez-Harms J, Vorobyev M, Schorn J*, et al.*** **2012**. Evidence of red sensitive photoreceptors in *Pygopleurus israelitus* (Glaphyridae: Coleoptera) and its implications for beetle pollination in the southeast Mediterranean. *Journal of Comparative Physiology A,* **198**: 451-463.

**McGregor RC.** **1899**. *Salvia Coccinea*, an ornithophilous plant. *The American Naturalist,* **33**: 953-955.

**Meerabai G.** **2013**. Evolutionary relationship of butterflies and their nectar flowers. *Indian Journal Applied and Pure Biology Letters,* **28**: 55-60.

**Mendonça LB, dos Anjos L.** **2005**. Hummingbirds (Aves, Trochilidae) and their flowers in an urban area of southern Brazil. *Revista Brasileira de Zoologia,* **22**: 51-59.

**Mendonça LB, dos Anjos L.** **2006**. Feeding behavior of hummingbirds and perching birds on *Erythrina speciosa* Andrews (Fabaceae) flowers in an urban area, Londrina, Paraná, Brazil. *Revista Brasileira de Zoologia,* **23**: 42-49.

**Micheneau C, Fournel J, Pailler T.** **2006**. Bird pollination in an angraecoid orchid on Reunion Island (Mascarene Archipelago, Indian Ocean). *Annals of Botany,* **97**: 965-974.

**Miller JAC, Henning L, Heazlewood VL*, et al.*** **2005**. Pollination biology of oilseed poppy, *Papaver somniferum* L. *Australian Journal of Agricultural Research,* **56**: 483-490.

**Miller RS, Nero RW.** **1983**. Hummingbird–sapsucker associations in northern climates. *Canadian Journal of Zoology,* **61**: 1540-1546.

**Mostafa NM, El-Dahshan O, Singab ANB.** **2013**. *Pyrostegia venusta* (Ker Gawl.) Miers: a botanical, pharmacological and phytochemical review. *Medicinal and Aromatic Plants,* **2**: 2-3.

**Nevling LIJ, Elias TS.** **1971**. *Calliandra haematocephala*: history, morphology, and taxonomy. *Journal of the Arnold Arboretum,* **52**: 69-85.

**Ng J, Smith SD.** **2016**. Widespread flower color convergence in Solanaceae via alternate biochemical pathways. *New Phytologist,* **209**: 407-417.

**Nicolson SW.** **2002**. Pollination by passerine birds: why are the nectars so dilute? *Comparative Biochemistry and Physiology B,* **131**: 645-652.

**Nilsson LA.** **1980**. The pollination ecology of *Dactylorhiza sambucina* (Orchidaceae). *Botaniska Notiser,* **133**: 367-385.

**Olesen JM, Alarcón M, Ehlers BK, Aldasoro JJ, Roquet C.** **2012**. Pollination, biogeography and phylogeny of oceanic island bellflowers (Campanulaceae). *Perspectives in Plant Ecology Evolution and Systematics,* **14**: 169-182.

**Ollerton J, Cranmer L, Stelzer RJ, Sullivan S, Chittka L.** **2009**. Bird pollination of Canary Island endemic plants. *Naturwissenschaften,* **96**: 221-232.

**Ortiz PL, Berjano R, Talavera M, Rodríguez-Zayas L, Arista M.** **2015**. Flower colour polymorphism in *Lysimachia arvensis*: how is the red morph maintained in Mediterranean environments? *Perspectives in Plant Ecology Evolution and Systematics,* **17**: 142-150.

**Peitsch D, Fietz A, Hertel H, de Souza J, Ventura DF, Menzel R.** **1992**. The spectral input systems of hymenopteran insects and their receptor-based colour vision. *Journal of Comparative Physiology A,* **170**: 23-40.

**Pickens AL.** **1936**. Steps in the development of the bird-flower. *Condor,* **38**: 150-154.

**Prendergast HDV.** **1982**. Pollination of *Hibiscus rosa-sinensis*. *Biotropica,* **14**: 287-287.

**Proctor M, Yeo P, Lack A.** **1996**. *The natural history of pollination.* London, UK.: Harper Collins.

**Quagliotti L, Marletto F.** **1987**. Research on the pollination of Runner Bean (*Phaseolus coccineus* L.) for dry grain production. *Advances in Horticultural Science,* **1**: 43-49.

**Rajaselvam RJ, Gunasena HPM, Wickramasinghe IP.** **1995**. Reproductive biology of *Calliandra calothyrsus* in relation to its seed production in Sri Lanka. *Tropical Agricultural Research,* **7**: 124-131.

**Rangaiah K, Purnachandra Rao S, Solomon Raju AJ.** **2004a**. Bird-pollination and fruiting phenology in *Spathodea campanulata* Beauv. (Bignoniaceae). *Beiträge zur Biologie der Pflanzen,* **73**: 395-408.

**Rangaiah K, Solomon Raju AJ.** **2017**. Reproductive ecology of some ornithophilous tropical tree species. *Advances in Pollen Spore Research,* **35**: 147-219.

**Rangaiah K, Solomon Raju AJ, Purnachandra Rao S.** **2004b**. Passerine bird-pollination in the Indian coral tree, *Erythrina variegata* var. *orientalis* (Fabaceae). *Current Science,* **87**: 736-739.

**Robertson C.** **1917**. Flowers and insects. XX. Evolution of entomophilous flowers. *Botanical Gazette,* **63**: 307-316.

**Rodrã­guez-Rodrã­guez MC, Valido A.** **2008**. Opportunistic nectar-feeding birds are effective pollinators of bird-flowers from Canary Islands: experimental evidence from *Isoplexis canariensis* (Scrophulariaceae). *American Journal of Botany,* **95**: 1408-1415.

**Rodríguez-Rodríguez MC, Valido A.** **2011**. Consequences of plant-pollinator and floral-herbivore interactions on the reproductive success of the Canary Islands endemic *Canarina canariensis* (Campanulaceae). *American Journal of Botany,* **98**: 1465-1474.

**Roubik DW, Holbrook NM, Parra GV.** **1985**. Roles of nectar robbers in reproduction of the tropical treelet *Quassia amara* (Simaroubaceae). *Oecologia,* **66**: 161-167.

**Saito N, Harborne JB.** **1992**. Correlations between anthocyanin type, pollinator and flower color in the Labiatae. *Phytochemistry,* **31**: 3009-3015.

**Sakai S, Kato M, Inoue T.** **1999**. Three pollination guilds and variation in floral characteristics of Bornean gingers (Zingiberaceae and Costaceae). *American Journal of Botany,* **86**: 646-658.

**Sakai S, Kawakita A, Ooi K, Inoue T.** **2013**. Variation in the strength of association among pollination systems and floral traits: evolutionary changes in the floral traits of Bornean gingers (Zingiberaceae). *American Journal of Botany,* **100**: 546-555.

**Sazima I, Buzato S, Sazima M.** **1995**. The saw-billed hermit *Ramphodon naevius* and its flowers in southeastern Brazil. *Journal für Ornithologie,* **136**: 195-206.

**Sazima I, Buzato S, Sazima M.** **1996**. An assemblage of hummingbird‐pollinated flowers in a montane forest in southeastern Brazil. *Plant Biology,* **109**: 149-160.

**Schemske DW.** **1976**. Pollinator specificity in *Lantana camara* and *L. trifolia* (Verbenaceae). *Biotropica,* **8**: 260-264.

**Schmidt-Lebuhn AN, Schwerdtfeger M, Kessler M, Lohaus G.** **2007**. Phylogenetic constraints vs. ecology in the nectar composition of Acanthaceae. *Flora: Morphology, Distribution, Functional Ecology of Plants,* **202**: 62-69.

**Scogin R.** **1988**. Floral anthocyanidins of bird-visited flowers. *Botanical Gazette,* **149**: 437-442.

**Scott-Elliot GF.** **1890**. Ornithophilous flowers in South Africa. *Annals of Botany,* **4**: 265-280.

**Silva BG, Piratelli AJ.** **2014**. Foraging behaviour of the scale-throated hermit *Phaethornis eurynome* Lesson, 1832 (Aves, Trochilidae) in *Vriesea incurvata* Gaudich (Bromeliaceae). *Brazilian Journal of Biology,* **74**: 311-314.

**Smith-Ramirez C.** **1993**. Los picaflores y su recurso floral en el bosque templado de la isla de Chiloé, Chile. *Revista Chilena de Historia Natural,* **66**: 65-73.

**Smith-Ramirez C, Armesto JJ.** **2010**. Foraging behaviour of bird pollinators on *Embothrium coccineum* (Proteaceae) trees in forest fragments and pastures in southern Chile. *Austral Ecology,* **28**: 53-60.

**Smith-Ramírez C, Martinez P, Nuñez M, González C, Armesto JJ.** **2005**. Diversity, flower visitation frequency and generalism of pollinators in temperate rain forests of Chiloe Island, Chile. *Botanical Journal of the Linnean Society,* **147**: 399-416.

**Smith SD, Hall SJ, Izquierdo PR, Baum DA.** **2008**. Comparative pollination biology of sympatric and allopatric Andean *Iochroma* (Solanaceae). *Annals of the Missouri Botanical Garden,* **95**: 600-617.

**Snow DW, Snow BK.** **1986**. Feeding ecology of hummingbirds in the Serra do Mar, southeastern Brazil. *El Hornero,* **12**: 286-296.

**Snow DW, Teixeira DL.** **1982**. Hummingbirds and their flowers in the coastal mountains of southeastern Brazil. *Journal Für Ornithologie,* **123**: 446-450.

**Solomon Raju AJ, Purnachandra Rao S, Zafar R, Roopkalpana P.** **2004**. Passerine bird-pollination and fruiting behaviour in *Erythrina variegata* L. (Fabaceae) in the Eastern Ghats forests, India. *Beiträge zur Biologie der Pflanzen,* **73**: 321-330.

**Stehmann JR.** **1987**. *Petunia exserta* (Solanaceae): Uma nova especie do Rio Grande do Sul, Brasil. *Napaea,* **2**: 19-21.

**Storti EF.** **2002**. Pollination biology and breeding system of *Passiflora coccinea* Aubl. in Manaus, Amazonas, Brazil. *Acta Amazonica,* **32**: 421-421.

**Streisfeld MA, Kohn JR.** **2007**. Environment and pollinator-mediated selection on parapatric floral races of *Mimulus aurantiacus*. *Journal of Evolutionary Biology,* **20**: 122-132.

**Subramanya S, Radhamani TR.** **1993**. Pollination by birds and bats. *Current Science,* **65**: 201-209.

**Taha E-KA, Taha RA, Al-Kahtani SN.** **2017**. Nectar and pollen sources for honeybees in Kafrelsheikh province of northern Egypt. *Saudi Journal of Biological Sciences*.

**Trelease W.** **1881**. The fertilization of *Salvia splendens* by birds. *The American Naturalist,* **15**: 265-269.

**Ueckermann C, van Rooyen MW.** **2000**. Insect pollination and seed set in four ephemeral plant species from Namaqualand. *South African Journal of Botany,* **66**: 28-30.

**Valdivia CE, Simonetti JA, Henríquez CA.** **2006**. Depressed pollination of *Lapageria rosea* Ruiz et Pav. (Philesiaceae) in the fragmented temperate rainforest of southern South America. *Biodiversity and Conservation,* **15**: 1845-1856.

**Valido A, Dupont YL, Olesen JM.** **2004**. Bird-flower interactions in the Macaronesian islands. *Journal of Biogeography,* **31**: 1945-1953.

**Van der Niet T, Cozien RJ, Johnson SD.** **2015**. Experimental evidence for specialized bird pollination in the endangered South African orchid *Satyrium rhodanthum* and analysis of associated floral traits. *Botanical Journal of the Linnean Society,* **177**: 141-150.

**Velasco Parreño SR.** **2016**. *Agroturism in the northwest of Pichincha province*, Universidad de Especialidades Turisticas, Quito, Ecuador.

**Villarreal MI.** **2016**. *Foraging patterns and population density of the buff-bellied hummingbird (Amazilia yucatanensis) in Hidalgo Xounty, TX,* MSc Thesis, University of Texas Rio Grande Valley, America.

**Vitali-Veiga MJ, Machado VLL.** **2000**. Visitantes florais de *Erythrina speciosa* Andr. (Leguminosae). *Revista Brasileira de Zoologia,* **17**: 369-383.

**Vizentin-Bugoni J, Maruyama PK, Sazima M.** **2014**. Processes entangling interactions in communities: forbidden links are more important than abundance in a hummingbird-plant network. *Proceedings Biological Sciences,* **281**: 86-108.

**Waser NM.** **1979**. Pollinator availability as a determinant of flowering time in ocotillo (*Fouquieria splendens*). *Oecologia,* **39**: 107-121.

**Webb CJ.** **1984**. Hummingbird pollination of *Malvaviscus arboreus* in Costa Rica. *New Zealand Journal of Botany,* **22**: 575-581.

**Wester P, Claßen-Bockhoff R.** **2007**. Floral diversity and pollen transfer mechanisms in bird-pollinated *Salvia* species. *Annals of Botany,* **100**: 401-421.

**Wester P, Claßen-Bockhoff R.** **2011**. Pollination syndromes of new world *Salvia* species with special reference to bird pollination. *Annals of the Missouri Botanical Garden,* **98**: 101-155.

**Willis EO.** **2002**. Birds at *Eucalyptus* and other flowers in Southern Brazil: A review. *Ararajuba,* **10**: 43-66.

**Wyatt R.** **1980**. The impact of nectar-robbing ants on the pollination system of *Asclepias curassavica*. *Bulletin of the Torrey Botanical Club,* **107**: 24-28.

**Yumoto T.** **1987**. Pollination systems in a warm temperate evergreen broad-leaved forest on Yaku Island. *Ecological Research,* **2**: 133-145.

**Zheng H, Zhang WW, Xu F, Zhang H, Chen XM, Rui YK.** **2013**. Study on volatile components of butterfly nectar plants and host plants. *Asian Journal of Chemistry,* **25**: 7861-7863.
