# Supplementary material for: Red flowers differ in shades between pollination systems and across continents
Source: Ann Bot. 2020 Jun 1;126(5):837–48. doi: 10.1093/aob/mcaa103 (PMC7539362; doi:10.1093/aob/mcaa103)
Supplement: mcaa103_suppl_Supplementary-Methods [file mcaa103_suppl_supplementary-methods.docx]

**Original Article**

**Red flowers differ in shades between pollination systems and across continents**

Zhe Chen^1,2†^, Yang Niu^1*†^, Chang-Qiu Liu^3^ and Hang Sun^1*^

**Methods S1 Methods for calculating photon catch and chromatic contrast in the CH and the RNL models.**

Photoreceptor photon catch of photoreceptor type $i$, resulting from the stimulation of floral reflectance, is calculated by:

$Q_{i}=\int_{300}^{700} I\left( \lambda\right)R\left( \lambda\right)S_{i}\left( \lambda\right)d\lambda$, (1)

where $I$ is the illuminant spectrum, for which we used the standard daylight D65 in the present study, $R$ is the reflectance spectrum of a stimulus (e.g. flowers), and $S_{i}$ is the spectral sensitivity of photoreceptor $i$. For bumblebees (*Bombus terrestris dalmatinus*), receptor sensitivity functions were obtained from Skorupski *et al.* (2007). For birds, average receptor sensitivity functions of UVS-birds and of VS-birds (ultraviolet and violet sensitive birds) based on the model from Endler and Mielke (2005) were used. In addition, receptor sensitivity functions of the hummingbird *Sephanoides sephaniodes* (Herrera *et al.*, 2008) were also used.

The photon catch is then normalized based on the photon catch from the background $Q_{back, i}$, following the von Kries transformation:

$q_{i}=Q_{i}{}/{Q_{back, i}}$, (2)

$Q_{back, i}$, as $Q_{i}$, is calculated based on formula (1), except that the floral reflectance $R$ is replaced by the leaf background reflectance $R_{back}$, for which we used an average of 230 leaf reflectances according to Chittka *et al.* (1994).

Then photon catch is transformed to the photoreceptor signal using a hyperbolic transformation (for the CH model, Chittka, 1992):

$s_{i}=\frac{q_{i}}{q_{i}+1}$, (3)

or a logarithmic transformation (for the RNL model, Vorobyev *et al.*, 2001):

$s_{i}=ln(q_{i})$. (4)

In the CH model (Chittka, 1992), chromatic contrast is calculated by:

$\Delta S= \sqrt{(x^{2}+y^{2})}$, (5)

Where $x$ and $y$ are coordinates of a colour locus in the CH colour space, which are determined by:

$\left\{ x, y \right\}=\left\{ \frac{\sqrt{3}}{2}\left( s_{3}-s_{1} \right); s_{2}-\frac{1}{2}(s_{3}+s_{1}) \right\}$. (6)

In the RNL model (Vorobyev *et al.*, 2001), the chromatic contrast for trichromatic colour vision is calculated by:

$\Delta S= \sqrt{\frac{{\omega_{1}}^{2}{(s_{3}-s_{2})}^{2} + {\omega_{2}}^{2}{(s_{3}-s_{1})}^{2}+ {\omega_{3}}^{2}{(s_{1}-s_{2})}^{2}}{\left( \omega_{1}\omega_{2} \right)^{2} + \left( \omega_{1}\omega_{3} \right)^{2} +\left( \omega_{2}\omega_{3} \right)^{2}}}$, (7)

and for tetrachromatic colour vision it is calculated by:

$\Delta S= \sqrt{\frac{\left( \omega_{1}\omega_{2} \right)^{2}{(s_{4}-s_{3})}^{2} + \left( \omega_{1}\omega_{3} \right)^{2}{(s_{4}-s_{2})}^{2} + \left( \omega_{1}\omega_{4} \right)^{2}{(s_{3}-s_{2})}^{2} +\left( \omega_{2}\omega_{3} \right)^{2}{(s_{4}-s_{1})}^{2} + \left( \omega_{2}\omega_{4} \right)^{2}{(s_{3}-s_{1})}^{2} + \left( \omega_{3}\omega_{4} \right)^{2}{(s_{2}-s_{1})}^{2}}{\left( \omega_{1}\omega_{2}\omega_{3} \right)^{2} + {\left( \omega_{1}\omega_{2}\omega_{4} \right)^{2}+\left( \omega_{1}\omega_{3}\omega_{4} \right)}^{2} +\left( \omega_{2}\omega_{3}\omega_{4} \right)^{2}}}$. (8)

$\omega_{i}$is the Weber fraction of receptor $i,$ which was used as a substitute for the noise value of photoreceptor channel $i$; as it refers to the minimum stimuli difference/change to be perceptible, and any difference smaller than the Weber fraction is unnoticeable and just perceived as noise. It is estimated by:

$\omega_{i}= {\nu_{i}}/{\sqrt{\eta_{i}}}$. (9)

where $\nu_{i}$ is the standard deviation of noise in a single cone, and $\eta_{i}$ refers to the relative abundance of receptor of type $i$. For bumblebees, 0.74, 0.67 and 0.61 were used as the Weber fractions for UV, blue and green receptors (Skorupski and Chittka, 2010). For birds, receptor abundance ratios (SWS1: SWS2: MWS: LWS) of 1:2:2:4 (for Pekin robin, *Leiothrix lutea*, Maier and Bowmaker, 1993) and 1:1:1:2 (for pigeon, *Columba livia*, Bowmaker *et al.*, 1997) were used as representatives for UVS- and VS-birds, respectively. Considering the known Weber fraction value 0.1 for the LWS photoreceptor of *Leiothrix lutea* (Maier, 1992), the corresponding Weber fraction values for both UVS-birds and the hummingbird were set to 0.2, 0.1414, 0.1414, and 0.1; for VS-birds, they were 0.1414, 0.1414, 0.1414 and 0.1.

**LITERATURE CITED**

**Bowmaker JK, Heath LA, Wilkie SE, Hunt DM.** **1997**. Visual pigments and oil droplets from six classes of photoreceptor in the retinas of birds. *Vision Research,* **37**: 2183-2194.

**Chittka L.** **1992**. The colour hexagon: a chromaticity diagram based on photoreceptor excitations as a generalized representation of colour opponency. *Journal of Comparative Physiology A,* **170**: 533-543.

**Chittka L, Shmida A, Troje N, Menzel R.** **1994**. Ultraviolet as a component of flower reflections, and the color perception of Hymenoptera. *Vision Research,* **34**: 1489-1508.

**Endler JA, Mielke PWJ.** **2005**. Comparing entire colour patterns as birds see them. *Biological Journal of the Linnean Society,* **86**: 405-431.

**Herrera G, Zagal JC, Diaz M*, et al.*** **2008**. Spectral sensitivities of photoreceptors and their role in colour discrimination in the green-backed firecrown hummingbird (*Sephanoides sephaniodes*). *Journal of Comparative Physiology A,* **194**: 785-794.

**Maier EJ.** **1992**. Spectral sensitivities including the ultraviolet of the passeriform bird *Leiothrix lutea*. *Journal of Comparative Physiology A,* **170**: 709-714.

**Maier EJ, Bowmaker JK.** **1993**. Colour vision in the passeriform bird, *Leiothrix lutea*: correlation of visual pigment absorbance and oil droplet transmission with spectral sensitivity. *Journal of Comparative Physiology A,* **172**: 295-301.

**Skorupski P, Chittka L.** **2010**. Differences in photoreceptor processing speed for chromatic and achromatic vision in the Bumblebee, *Bombus terrestris*. *Journal of Neuroscience,* **30**: 3896-3903.

**Skorupski P, Döring TF, Chittka L.** **2007**. Photoreceptor spectral sensitivity in island and mainland populations of the bumblebee, *Bombus terrestris*. *Journal of Comparative Physiology A,* **193**: 485-494.

**Vorobyev M, Brandt R, Peitsch D, Laughlin SB, Menzel R.** **2001**. Colour thresholds and receptor noise: behaviour and physiology compared. *Vision Research,* **41**: 639-653.
